# Social Physiology: Human homeostasis is more efficient in social proximity

**DOI:** 10.1126/sciadv.aeg4937

**Published:** 2026-09-23

**Authors:** Monia Masalha, Matan Cohen, Maayan Aloni, Einav Toledo, Ella Stahl, Diane Shachar, Or Eliav, Shai Fuchs, Shir Atzil

**Affiliations:** ^1^ Department of Psychology, The Hebrew University of Jerusalem, Jerusalem, Israel.; ^2^ Paediatric Endocrine and Diabetes Unit, Edmond and Lily Safra Children's Hospital, Sheba Medical Center, Ramat Gan, Israel.

## Abstract

Physiological homeostasis is traditionally viewed as an individual process optimized through evolution to minimize the energetic costs of regulation. However, it remains unknown whether social proximity enhances the efficiency of homeostatic regulation, including in systems not traditionally viewed as socially sensitive. Glucose metabolism is one such system. In a set of preregistered experiments, we found that glucose homeostasis following a carbohydrate challenge is more efficient in a Social condition than an Alone condition, exhibiting lower perturbation amplitude and faster recovery (*n* = 108). The social benefit in homeostasis replicated in thermoregulation during a cold challenge (*n* = 116) and in sympathetic regulation during stress in adults (*n* = 115) and infants (*n* = 56). These findings demonstrate Social Physiology, a cross-domain principle whereby physiological homeostasis is more efficient in social proximity. Evolutionarily, Social Physiology reveals a psychobiological benefit of sociality, beyond its behavioral and ecological advantages, that may contribute to the emergence and maintenance of social bonds.

## INTRODUCTION

Energy efficiency is an organizing principle of life and evolution (*1*–*4*). In a living organism, adaptations that improve physiological regulation reduce the metabolic burden and free resources that can be allocated to other vital functions (*5*, *6*) [for discussion, see the work of Theriault *et al.* (*6*)]. Over time, the efficiency of these regulatory processes sustains physical and mental health (*7*–*9*). One of the hallmark evolutionary adaptations in humans and other mammals is sociality, raising a fundamental question: Given strong positive selection and convergent evolution across distantly related taxa (e.g., mammals, birds, and insects), might it serve not only behavioral and ecological functions (e.g., cooperation, threat buffering, and reproduction) but also a deeper biological imperative—enhancing the efficiency of homeostasis regulation? On this account, the putative function of sociality should extend beyond reproduction or stress to encompass canonical homeostatic systems typically regarded as internally maintained and individually controlled.

Here, we propose and test the hypothesis that maintaining physiological homeostasis is more efficient in a social proximity, yielding immediate Social Physiological Gains. These gains reflect improved efficiency of homeostatic regulation itself, even in physiological systems not traditionally considered socially regulated. A rigorous proof-of-concept test for this hypothesis in humans is a physiological system that is metabolically costly, autonomic, not intentionally controlled, and not traditionally conceptualized as socially responsive, such as glucose homeostasis (*10*). In this research, participants completed a standardized carbohydrate challenge (*11*) in an Alone condition compared to a Social condition with a close partner to test whether social proximity improves the efficiency of glucose homeostasis by attenuating the magnitude of response perturbation and accelerating the return to the baseline. We then assessed the cross-domain scope of this effect by replicating the same design to thermoregulation and autonomic arousal, asking whether social proximity similarly enhances homeostatic efficiency across systems. These systems were selected because they represent core, energetically costly homeostatic processes governing energy expenditure and autonomic control and rely on distinct physiological pathways (*4*, *12*). We predicted that physiological homeostasis would be more efficient in the Social condition compared to the Alone condition and that this effect would replicate across all measured systems. The experiments were preregistered at https://osf.io/.

## RESULTS

### Quantifying Social Physiology

Homeostatic systems must continuously meet regulatory demands under energetic constraints (*13*). Homeostatic regulation optimizes the balance between mounting adaptive physiological responses and limiting unnecessary physiological expenditure (*13*–*21*). This balance is implemented through negative feedback control processes that attenuate physiological activity once a need has been addressed, thereby constraining excessive energetic costs (*3*, *13*, *15*, *21*–*23*). These dynamics were quantified using the incremental area under the curve (iAUC) of the physiological response, which indexes the cumulative magnitude and duration of the physiological perturbation from the baseline over time for a given challenge (*23*, *24*). Efficiency reflects the extent to which regulatory demands are met with minimal overall physiological expenditure (*23*, *24*). Accordingly, when the physiological challenge is held constant across conditions, a lower iAUC reflects lower cumulative physiological expenditure associated with resolving that challenge (*13*–*19*, *23*). This approach enables a unified quantification of homeostatic dynamics across systems by integrating both perturbation amplitude and recovery dynamics. Focusing on the shared regulatory features makes this approach domain-general while accommodating differences in how efficiency is expressed across physiological systems, whether through reduced perturbation magnitude, faster recovery, or both.

**Fig. 1.**
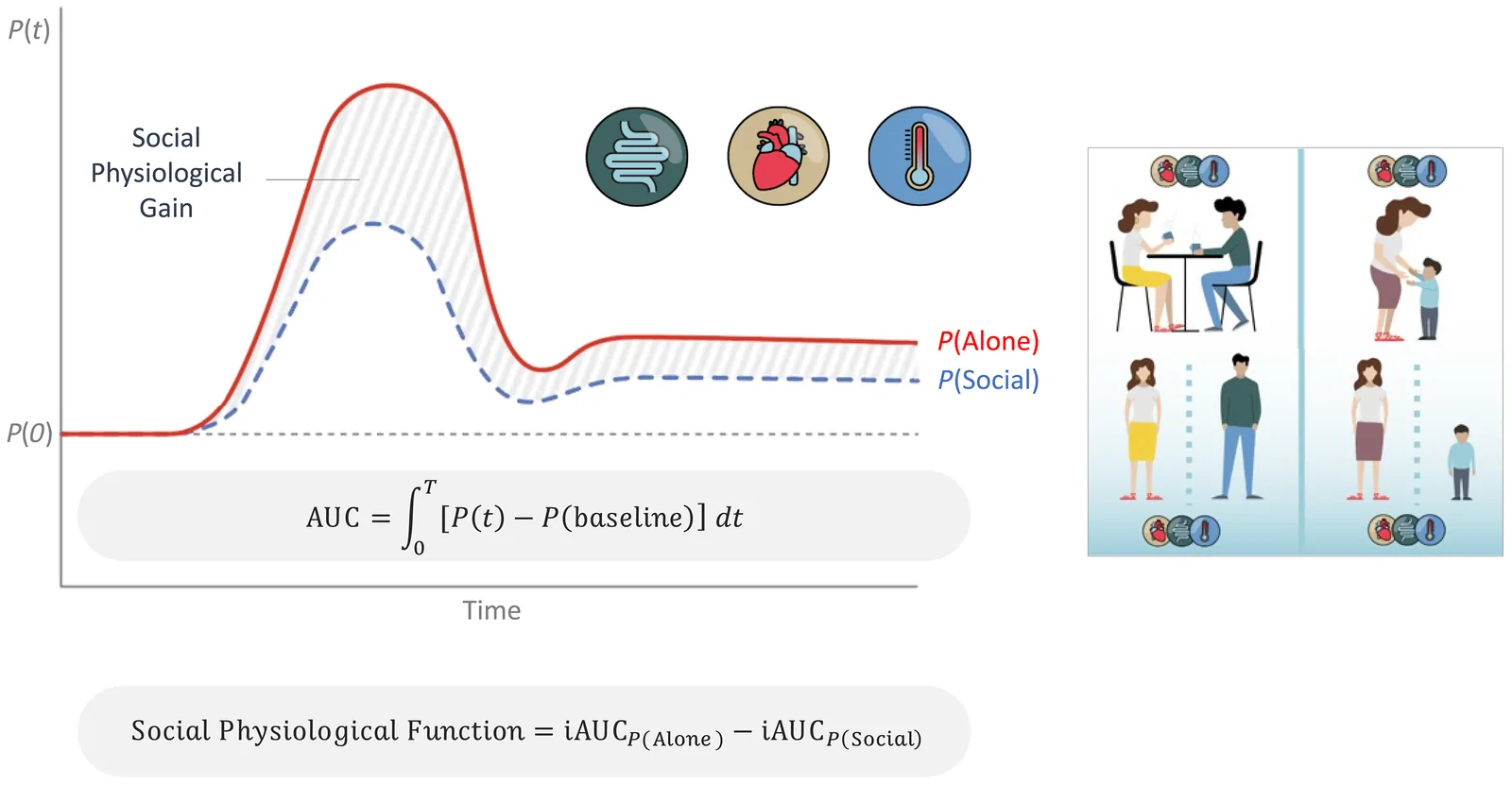
Quantifying the effect of social proximity on physiological homeostasis. Homeostatic regulatory dynamics were quantified using the iAUC, computed as the integral of the deviation of a physiological signal *P*(*t*) from the baseline over the challenge window (*T*), capturing both the magnitude and duration of the response: $\text{AUC}=\int_{0}^{T}\left[P\left(t\right)-P\left(\text{baseline}\right)\right]\;\mathrm{dt}$. Larger iAUC values indicate a larger and/or more prolonged physiological response deployed to meet a regulatory demand and, consequently, greater physiological effort (*13*, *15*, *21*–*23*). When the physiological challenge is held constant, lower iAUC values reflect that the physiological demand was met with lower overall expenditure (*13*–*18*, *23*). The Social Physiological Gain is the directional difference in iAUC between the Social and Alone conditions during the challenge: $\text{Social Physiological Function}={\text{iAUC}}_{P\left(\text{Alone}\right)}-{\text{iAUC}}_{P\left(\text{Social}\right)}$. Social Physiological Gain values indicate that the same physiological challenge was resolved with lower overall physiological expenditure in the Social condition, reflecting greater regulatory efficiency. This was replicated across three homeostatic systems: glucose metabolism, thermoregulation, and sympathetic arousal. The Social condition was operationalized as the presence of a close partner (a romantic partner in the adult experiments and a mother in the infant experiment) to maximize the likelihood of physiologically relevant social regulation to establish a proof of concept. Graphic illustrations were created by Y. Yadegari from HandMed.

Social Physiology refers to the extent to which physiological regulation operates more efficiently in a social context compared to being alone. For a given physiological challenge held constant across conditions, we predicted that iAUC would be reduced in the Social condition relative to the Alone condition, reflecting either attenuated perturbation amplitude, faster restoration toward the baseline, or both (Fig. 1). The Social Physiology effect was assessed using within-participant and between-participant analyses. The within-participant approach, with a counterbalanced order, afforded an individual estimate of social effects by allowing each individual to serve as their own control. The between-participants design mitigated potential carryover from repeated physiological challenges and tested whether the social advantage generalized to independent groups. This design was replicated across three homeostatic systems: glucose metabolism, thermoregulation, and sympathetic arousal.

### Human glucose metabolism is more efficient in social proximity than alone

**Fig. 2.**
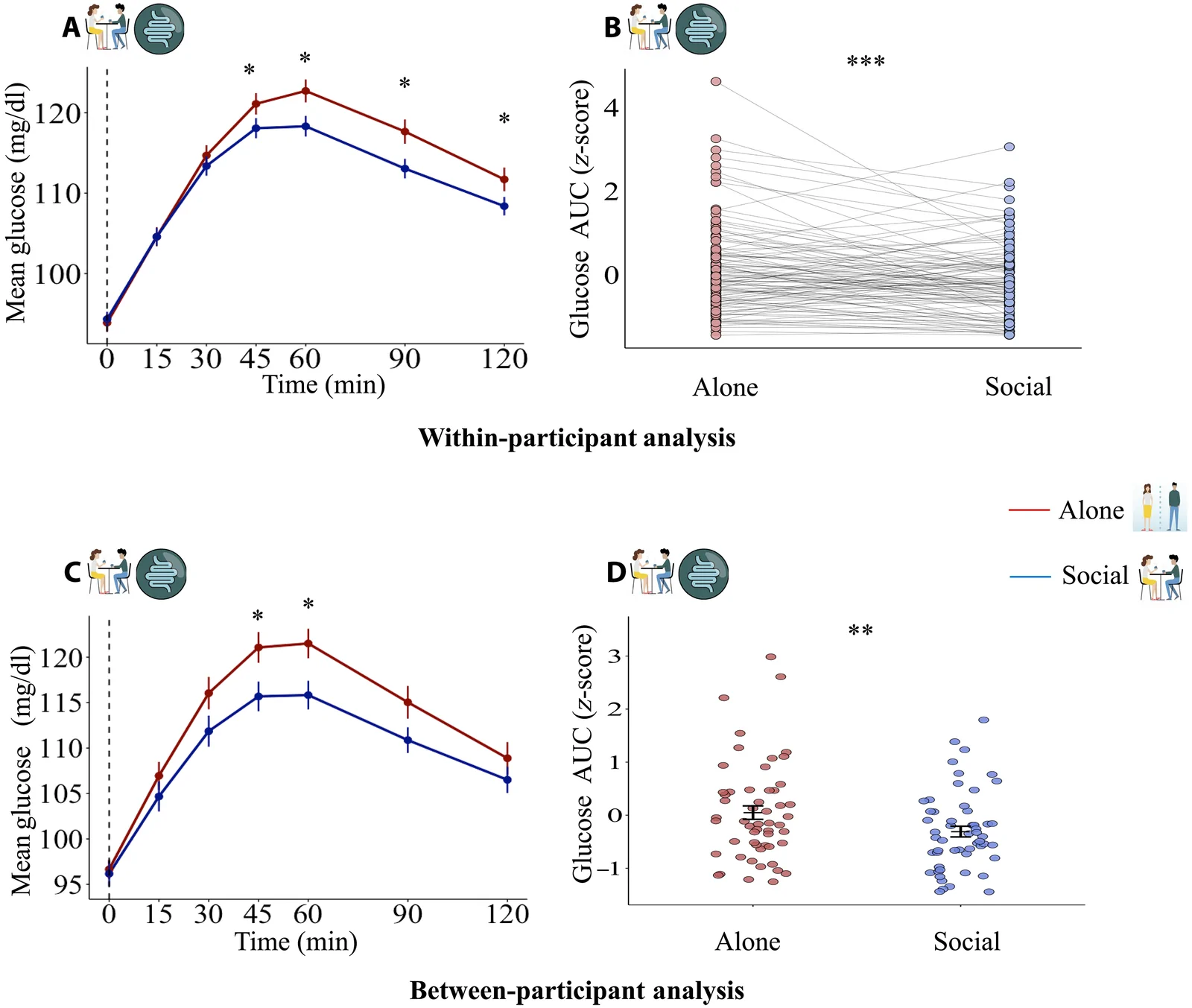
Glucose homeostasis is more efficient in a social context than alone. Postprandial glucose regulation was more efficient in the Social condition (blue) than in the Alone condition (red), with reduced perturbation amplitude and accelerated recovery, yielding lower iAUC. Dashed lines indicate the time point at which carbohydrate consumption ended. Curves represent the group means ± SEM; black bars indicate the condition means ± SEM. (**A**) The postprandial glycemic response is attenuated and recovers more rapidly in the Social condition than in the Alone condition (within-participant analysis). (**B**) The mean iAUC is lower in the Social condition than in the Alone condition (within participants; *d* = 0.30; *P* = 0.001; *n* = 108). Gray lines connect within-individual paired observations across conditions. (**C**) The postprandial glycemic response is attenuated and recovers more rapidly in the Social condition than in the Alone condition (between-participant analysis). (**D**) The mean iAUC is lower in the Social condition than in the Alone condition (between participants; *d* = 0.42; *P* = 0.01; *n*_(Alone)_ = 54, *n*_(Social)_ = 54). *P ≤ 0.05; **P ≤ 0.01; ***P ≤ 0.001. Graphic illustrations were created by Y. Yadegari from HandMed.

The experiment consisted of a standardized carbohydrate-meal test, in which participants consumed 50 g of carbohydrates by eating 102 g of a standardized white-wheat bread (*11*, *25*, *26*), and their postprandial glycemic response was sampled over 120 min (*26*). Postprandial plasma glucose levels showed reduced perturbation amplitude and accelerated recovery in the Social condition relative to the Alone condition, yielding a lower iAUC [within-participant: Alone area under the curve (AUC): mean = 1926.51 mg/dl·min; SE = 127.01 mg/dl·min; Social AUC: mean = 1576.64 mg/dl·min; SE = 102.77 mg/dl·min; paired-samples *t*(107) = 3.1; *d* = 0.30; *P* = 0.001; a 14.55% mean reduction in the Social versus Alone conditions; *n* = 108; between-participant: Alone AUC: mean = 1810.19 mg/dl·min; SE = 153.72 mg/dl·min; *n* = 54; Social AUC: mean = 1377.48 mg/dl·min; SE = 121.96 mg/dl·min; *n* = 54; Welch *t*(100.79) = 2.21; *d* = 0.42; *P* = 0.01; a 23.9% mean reduction in the Social versus Alone conditions]. Detailed amplitude and recovery analyses are provided in section S1.2. Glucose levels were comparable across conditions during the baseline and diverged following the meal, with lower glucose levels in the Social condition at all postprandial time points. In the within-participant analyses, glucose levels were lower in the Social condition at all postprandial time points {*T*_45_ [*t*(107) = 1.81; *P* = 0.07 (one-tailed *P* = 0.04)], *T*_60_ [*t*(107) = 3.17; *P* = 0.002 (one-tailed *P* = 0.001)], *T*_90_ [*t*(107) = 2.49; *P* = 0.01 (one-tailed *P* = 0.007)], and *T*_120_ [*t*(107) = 2.13; *P* = 0.04 (one-tailed *P* = 0.02)]}. A similar pattern emerged in the between-participant analyses, with lower glucose levels in the Social condition at *T*_45_ and *T*_60_ {*T*_45_ [*t*(106) = 1.91; *P* = 0.06 (one-tailed *P* = 0.03)], *T*_60_ [*t*(104.9) = 2.38; *P* = 0.02 (one-tailed *P* = 0.01)], *T*_90_ [*t*(101.1) = 1.35; *P* = 0.18 (one-tailed *P* = 0.09)], and *T*_120_ [*t*(101) = 1.04; *P* = 0.3 (one-tailed *P* = 0.15)]}. These findings show that glucose homeostasis operates more efficiently in a social context, both within individuals and across groups (Fig. 2).

### Social Physiology is consistent across physiological systems and ages

**Fig. 3.**
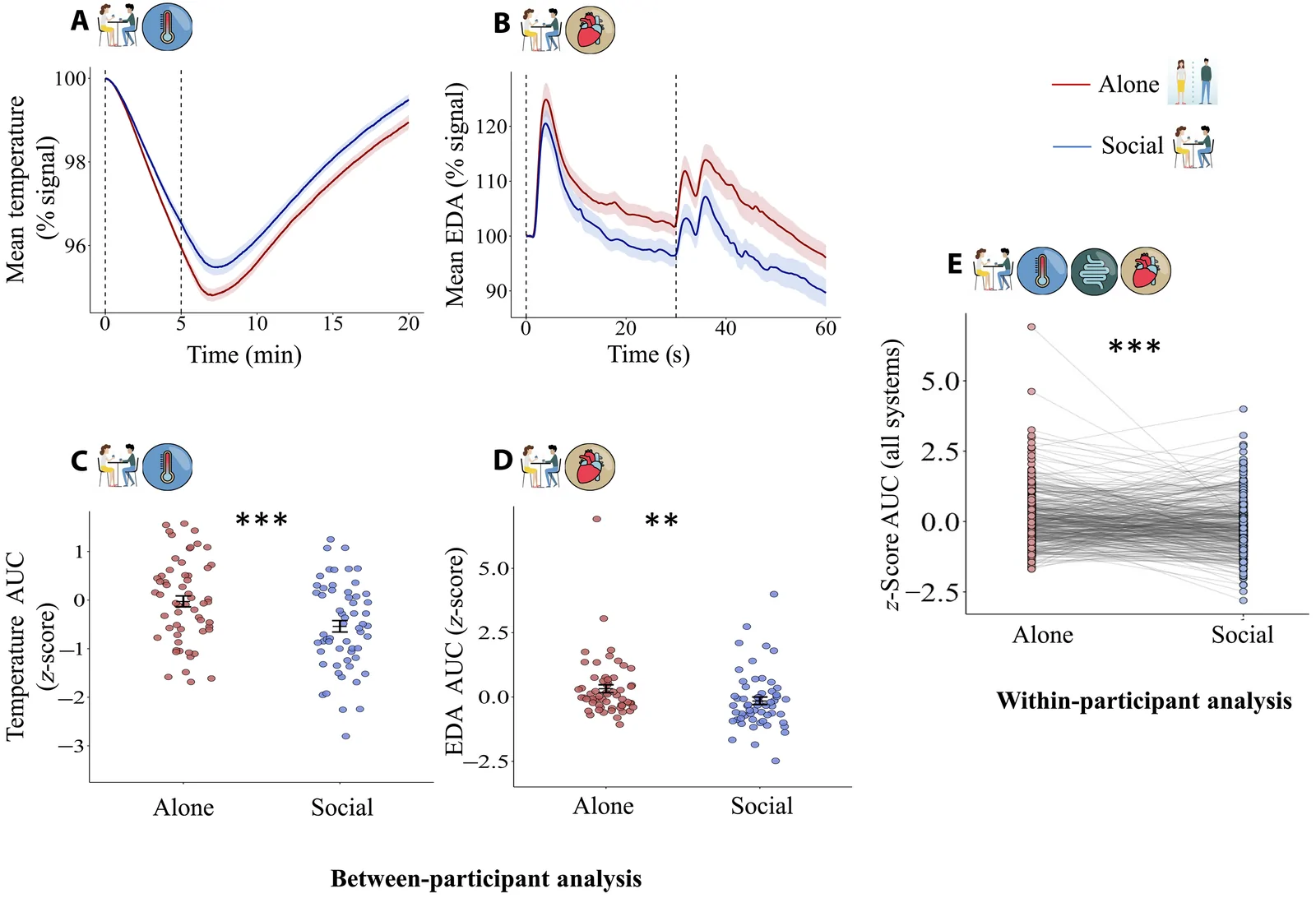
Homeostasis regulation is consistently more efficient in a social context than alone across physiological systems. Across systems, homeostasis regulation is more efficient in the Social condition (blue) than in the Alone condition (red). (**A**) The thermoregulatory response to the cold challenge is milder in the Social condition. Dashed lines indicate cold-room entry and exit. Curves represent the group means ± SEM. (**B**) The sympathetic response to an electric shock is lower in the Social condition. Dashed lines indicate shock administration. Curves represent the group means ± SEM. (**C**) The mean temperature iAUC (°C·min) is lower in the Social condition than in the Alone condition (between participants; *P* = 0.001; *d* = 0.59; *n*_(Alone)_ = 57, *n*_(Social)_ = 58). Points represent participants; black bars indicate the means ± SEM. (**D**) The mean sympathetic iAUC (μS·s) is lower in the Social condition than in the Alone condition (between participants; *P* = 0.01; *d* = 0.41; *n*_(Alone)_ = 57, *n*_(Social)_ = 58). Points represent participants; black bars indicate the means ± SEM. (**E**) Main effect of Social Physiology across all adult systems, with lower iAUC in the Social condition than in the Alone condition (within-participant linear mixed-effects model: β = 0.11, *P* = 0.001; *n* = 339 observations across glucose, temperature, and EDA). Gray lines connect within-individual paired observations across conditions. *P ≤ 0.05; **P ≤ 0.01; ***P ≤ 0.001. Graphic illustrations were created by Y. Yadegari from HandMed.

The Social Physiology effect was consistently observed across all tested physiological systems. In particular, it was demonstrated in two additional homeostatic systems in adults, thermoregulation during a cold challenge and sympathetic regulation during a stress challenge, and in sympathetic regulation during stress in infants. Robust across systems and age groups, the Social condition produced more efficient homeostasis regulation, quantified by lower iAUC in the Social Condition compared to the Alone condition (Fig. 3).

### Human thermoregulation is more efficient in a social context than alone

The experiment consisted of a cold challenge, in which participants entered a cold room at 12°C for 5 min, followed by a 15-min recovery period at 25°C, and their skin temperature was sampled at 1 Hz for 20 min. Temperature levels showed reduced perturbation amplitude and accelerated recovery in the Social condition relative to the Alone condition, yielding a lower iAUC [within-participant: Alone AUC: mean = 3712.79°C·min; SE = 96.86°C·min; Social AUC: mean = 3460.14°C·min; SE = 110.24°C·min; paired-samples *t*(115) = 1.84; *d* = 0.17; *P* = 0.03; a 6.76% mean reduction in the Social versus Alone condition; *n* = 116 (fig. S1); between-participant: Alone AUC: mean = 3556.96°C·min; SE = 126.11°C·min; *n*_(Alone)_ = 58; Social AUC: mean = 2981.80°C·min; SE = 131.90°C·min; *n*_(Social)_ = 58; Welch *t*(113.77) = 3.15; *d* = 0.59; *P* = 0.001; a 16.17% mean reduction in the Social versus Alone conditions] (Fig. 3, A and C).

### Human sympathetic regulation in adults is more efficient in a social context than alone

The experiment consisted of a stress challenge with two mild electric shocks (an amplitude of 7 V and a duration of 10 ms) delivered to the forearm during a 1-min “stress period,” and electrodermal activity (EDA) was sampled at 2000 Hz over 60 s. EDA refers to the changes in the skin’s electrical conductance driven by eccrine sweat gland activity under sympathetic control and serves as an index of physiological arousal and stress (*27*–*30*). In adults, sympathetic arousal levels showed reduced perturbation amplitude and accelerated recovery in the Social condition relative to the Alone condition. This effect was significant in a between-participant analysis and trending toward significance in a within-participant analysis [within-participant: Alone AUC: mean = 6181.91 μS·s; SE = 76.23 μS·s; Social AUC: mean = 6058.19 μS·s; SE = 75.52 μS·s; paired-samples *t*(114) = 1.36; *d* = 0.13; *P* = 0.09; a 1.89% mean reduction in physiological cost in the Social versus Alone condition; *n* = 115 (fig. S1); between-participant: Alone AUC: mean = 6381.88 μS·s; SE = 124.84 μS·s; *n*_(Alone)_ = 57; Social AUC: mean = 5999.94 μS·s; SE = 119.66 μS·s; *n*_(Social)_ = 58; Welch *t*(112.87) = 2.21; *d* = 0.41; *P* = 0.01; a 5.98% mean reduction in the Social versus Alone conditions] (Fig. 3, B and D).

### Main effect of Social Physiology across three homeostatic systems in adults

To estimate the generalizability of Social Physiology across physiological systems, we *z*-normalized the iAUC values within each system to place glucose, temperature, and arousal on a common scale, enabling a pooled main-effect estimate of adult homeostasis. All models accounted for repeated observations within individuals and for dyadic dependencies (see “Participants” and “Statistical analyses” in Materials and Methods). Across all three preregistered adult experiments, a linear mixed-effects model showed a robust Social Physiology effect with significantly lower iAUC in the Social condition than in the Alone condition [within-participant: β = 0.11; *P* = 0.002 (one-tailed *P* = 0.0**0**1); a 7.59% mean reduction in the Social versus Alone condition; *n* = 339 observations with 180 adults; between-participant: β = 0.23; *P* < 0.001; a 12.07% mean reduction in the Social versus Alone condition; *n*_(Alone)_ = 170, *n*_(Social)_ = 169] (Fig. 3E). The Social Physiology effect remained robust following exclusion of outliers [>±3 SD; 4 of 678 data points; β = 0.11; *P* = 0.004 (one-tailed *P* = 0.002); detailed analyses are provided in section S1.3]. Moreover, the Social Physiology effect remained significant after controlling for relationship satisfaction [β = 0.11; *P* = 0.002 (one-tailed *P* = 0.001); 95% confidence interval (CI) [0.04, 0.18]; *n* = 337; detailed analyses are provided in section S1.4].

Together, these findings confirm that physiological processes are more efficient in a social context compared to alone. Social proximity influenced shared features of homeostatic regulation across physiologically distinct systems, suggesting a generalizable effect of Social Physiology.

### Human sympathetic regulation in infants is more efficient in a social context than alone

The infant experiment consisted of a stress challenge with an aversive auditory stimulus (an amplitude of 82 dB and a duration of 2 s), and EDA was sampled at 4 Hz for 10 s before and after the stimulus (overall 22 s). Sympathetic arousal levels in infants showed reduced perturbation amplitude and accelerated recovery in the Social condition relative to the Alone condition, yielding a lower iAUC [within-participant: Alone AUC: mean = 2265.35 μS·s; SE = 38.94 μS·s; Social AUC: mean = 2188.24 μS·s; SE = 17.5 μS·s; paired-samples *t*(55) = 1.75; *P* = 0.04; *d* = 0.23; a 2.55% mean reduction in the Social versus Alone condition; *n* = 56; between-participant: Alone AUC: mean =2272.87 μS·s; SE = 31.12 μS·s; *n*(Alone) = 26; Social AUC: mean =2176.4 μS·s; SE = 22.64 μS·s; *n*(Social) = 30; Welch *t*(47.09) = 2.51; *P* = 0.008; *d* = 0.68; a 4.24% mean reduction in the Social versus Alone condition] (fig. S1). The Social Physiological Gain was not correlated with infant age; detailed analyses are provided in section S1.5.

### Social Physiology is independent from physiological stress

To examine whether Social Physiological Gains in the Glucose Experiment reflect stress reduction in the presence of a familiar partner, we tested whether stress mediated the observed effects. Stress was assessed with EDA, a validated index of stress (*29*), continuously throughout the 2-hour postprandial period. EDA did not increase during the glucose challenge. Rather, EDA was lower during the challenge than at the baseline [baseline: mean = 9.39 μS; SD = 3.77; mean = 8.28 μS; SD = 3.62; *t*(103) = 4.07, *P* < 0.001 (two-tailed)]. Consistent with this, EDA levels during the challenge were not correlated with glucose AUC (Alone condition: *r* = −0.02; *P* = 0.81; BF_01_ = 4.31; Social condition: *r* = 0.07; *P* = 0.45; BF_01_ = 3.40; *n* = 104). Moreover, EDA levels did not differ between the Alone and Social conditions [Alone condition: mean = 8.28 μS; SD = 3.62; Social condition: mean = 8.80 μS; SD = 4.20; *t*(103) = −1.52; *P* = 0.13; BF_01_ = 3.02; *n* = 104]. Last, after controlling for EDA, the Social Physiology effect remained significant [β = 0.15; *P* = 0.002 (one-tailed *P* = 0.001); 95% CI = [0.05, 0.24]; *n* = 104]. Together, these findings indicate that the glucose challenge was not associated with elevated physiological stress and that the homeostatic advantage observed in the Social condition was independent of physiological stress. This suggests that the Social Physiology effect in the Glucose metabolism was not explained by stress reduction, as measured with EDA.

A similar trend was observed in the Temperature Experiment; however, these findings should be interpreted cautiously, as EDA measurement may have been less sensitive in the cold environment. Detailed analyses are provided in section S2. EDA could not be evaluated as a mediator in the Stress Experiment because it served as the primary outcome measure.

### Social Physiology is not reliably explained by physical touch

To examine whether the observed effects were driven by physical contact, we assessed the contribution of touch across experiments. In the Temperature Experiment, greater touch was associated with lower-temperature AUC in the Social condition, suggesting that touch may contribute to thermoregulation (*r* = −0.19; *d* = −0.39; *P* = 0.04; *n* = 113). No association between touch and physiological AUC was observed in the Glucose or Stress Experiments. Nevertheless, the Social Physiology effect remained evident after adjustment for touch across systems (β = 0.10; *P* = 0.005; one-tailed *P* = 0.002; 95% CI [0.03, 0.18]; *n* = 330) and in the Glucose Experiment [β = 0.14; *P* = 0.004 (one-tailed *P* = 0.002); 95% CI [0.05, 0.23]; *n* = 104]. In the Temperature Experiment, the effect remained significant under the preregistered one-tailed test (β = 0.10; *P* = 0.09; one-tailed *P* = 0.047; 95% CI [−0.02, 0.23]; *n* = 113). These findings suggest that touch may contribute to thermoregulation but is unlikely to be a reliable mediator of the Social Physiology effect. Further research is needed to determine how and under which contextual conditions touch contributes to Social Physiology. Detailed analyses examining region-specific touch are provided in the Supplementary Materials (section S3 for touch quantification and section S4 for sex-stratified analyses).

### Social Physiology persists after controlling for methodological confounds

We tested the robustness of the Social Physiology effect considering a range of potential methodological confounds, including condition order, experiment start time, and body mass index (across all experiments); eating duration and hemoglobin A1c (HbA1c) levels, which reflect the long-term glucose levels over the past 3 months (in the Glucose Experiment) (*31*); and room temperature fluctuations (in the Temperature Experiment). The Social Physiology effects remained significant after accounting for all the methodological confounds. Detailed analyses are provided in section S4.

## DISCUSSION

The study reveals a robust phenomenon in human physiology: Homeostasis regulation is more efficient in social proximity. Glucose regulation, an autonomic process not typically conceptualized as socially modulated, was more efficient in social proximity, independent of touch or stress. This effect generalized across all tested homeostatic systems, including glucose metabolism, thermoregulation, and sympathetic arousal in adults, as well as sympathetic arousal in infants. The consistent improvement in homeostatic efficiency across systems and ages provides convergent support for Social Physiology, indicating that in social animals such as humans, homeostasis is not solely an individual process but can be socially enhanced. Social Physiology reframes the biological benefits of sociality: Companionship is not only advantageous for protection, collaboration, or reproduction but can serve the fundamental function of enhancing homeostasis itself.

This discovery has two key implications; the first concerns evolution. Although attachment theory and social neuroscience have provided valuable insights into how close relationships are formed and maintained (*32*–*34*), they do not resolve a central paradox: Why are social animals motivated to invest considerable cognitive and physical effort (*35*–*37*) and accept risks of competition, conflict, and aggression (*38*, *39*) to engage in social interactions? The well-documented benefits of sociality are generally long-term (*40*–*42*) and do not account for the proximate motivation to seek social contact (*43*). Addressing this discrepancy requires identifying immediate gains that make social interaction worthwhile in the moment yet can also accumulate into long-term fitness benefits. Social Physiology offers an evolutionary account of how homeostatic efficiency could have shaped the emergence of sociality: Organisms whose homeostasis regulation is more efficient in social proximity would gain a fitness advantage, systematically favoring socially embedded individuals and thereby rendering sociality a target of positive selection. In this view, Social Physiology helps align proximate incentives for social contact (i.e., immediate physiological gains) with ultimate fitness benefits (i.e., physiological gains that can accumulate over time), supporting the emergence and maintenance of social bonds. More broadly, Social Physiology reframes regulation in social animals from autonomous individuals toward physiologically interdependent organisms (*32*, *44*, *45*) who are highly cooperative (*46*–*48*).

The second implication concerns health. While social bonds are widely associated with improved health, well-being, and longevity (*49*–*53*), their benefits have been primarily supported by epidemiological correlations. In particular, individuals with stronger ties exhibit better physical and mental health (*36*, *49*–*51*), greater longevity (*40*, *41*, *52*), enhanced productivity (*32*, *34*, *42*), and higher subjective well-being (*32*, *33*, *53*). Low social connectedness is associated with increased metabolic disorders (*54*–*58*), cardiovascular disease (*59*, *60*), cancer (*61*), slower wound healing (*60*), reduced sleep efficiency (*60*), morbidity, and mortality (*52*, *62*). In nonhuman social animals, social isolation is a robust maladaptive predictor. Experimental isolation shortens life span in *Drosophila melanogaster* (*63*), increases obesity and type 2 diabetes in mice (*64*), suppresses lymphocyte proliferation in pigs (*65*), and amplifies adrenomedullary and hypothalamic-pituitary-adrenal-axis stress activation in rats, pigs, and squirrel monkeys (*66*, *67*). These findings underscore the broad physiological scope of social influences across distinct regulatory domains. With specific regard to metabolism, findings from the social isolation literature identify multiple candidate mechanisms linking sociality and homeostasis, including central regulation, endocrine signaling, cellular energetics, temporal organization, thermoregulatory efficiency, and behavior. In mice, social isolation alters hypothalamic metabolic markers, reduces insulin signaling (*64*), impairs mitochondrial function (*68*), disrupts circadian and thermoregulatory processes (*69*), and promotes hyperphagia and obesity (*64*). Consistent with these findings, in humans, loneliness is associated with dysregulated glycemic control and predicts type 2 diabetes (*20*, *70*, *71*). While prior work has focused on the outcomes of social isolation, the present study identifies a direct, quantifiable benefit of social proximity in glucose metabolism, with convergent effects across additional homeostatic systems related to metabolic health. Social Physiology is not simply the inverse of social isolation. Rather, it addresses a distinct level of analysis and temporal resolution: Whereas epidemiological and isolation research has established ultimate associations between sociality and long-term health outcomes, Social Physiology quantifies the proximate benefits of social proximity at the level of moment-to-moment homeostatic regulation, thereby providing a candidate mechanistic pathway linking sociality to health and longevity.

Social Physiology may unfold through multiple, partially overlapping channels. One possibility is biophysical co-regulation, in which bodies directly influence each other through radiant and convective heat exchange (*72*–*77*) or through touch-evoked autonomic regulation (*72*–*74*). Physical touch contributed to thermoregulation. Nevertheless, touch was not a reliable mediator of the Social Physiology effects across experiments, including in the thermoregulation challenge, where such an association might be most expected. These findings suggest that although touch may contribute to social regulation, it is unlikely to be the primary driver of the effect.

A second, well-established pathway is a threat-buffering pathway grounded in Social Baseline Theory (*78*, *79*), which posits that the brain is calibrated to expect social proximity as a default condition (*80*, *81*). In this framework, companionship attenuates stress (*33*) and pain (*82*) and therefore frees physiological and psychological resources (*32*, *33*, *71*, *78*, *79*). Similarly, Attachment Theory suggests that relationships serve as a critical resource for emotion regulation, with a sense of attachment security providing resilience under stress (*83*–*86*). Consistent with this view, social isolation imposes threat (*71*, *78*), with downstream consequences for eating (*87*, *88*), glucose (*89*), and endocrine signaling (*90*–*92*), positioning metabolic regulation as secondary to social threat buffering (*93*–*95*). Social Baseline Theory and related accounts have compellingly highlighted the benefits of stress buffering, and the stress experiments in the present work replicate these established findings. Stress attenuation is a well-established contributor to social regulation (*32*, *33*, *71*). At the same time, the effects observed here suggest that the consequences of social buffering may extend beyond stress regulation, given that Social Physiology was evident in glucose regulation, a domain not typically considered a direct target of social support, and persisted after accounting for physiological stress. The glucose challenge did not produce elevated EDA, nor did EDA differ between the Social and Alone conditions, indicating that the task was not associated with heightened physiological stress. Nevertheless, social proximity improved glucose regulation. These findings suggest that Social Physiological Gains are not contingent on reductions in physiological stress and may extend beyond mechanisms traditionally associated with social support. Future research incorporating additional psychological, hormonal, and autonomic measures are important for clarifying the contribution of stress-related processes and the extent to which they interact with the effects observed here.

A third perspective concerns socially informed homeostatic control, or allostasis (*48*, *96*–*98*). In allostasis, prior knowledge is applied to predict and prepare for upcoming physiological demands (*18*, *99*–*101*). Social animals are born and develop in social proximity, and social input becomes integrated into the brain’s predictive models (*48*). This may render allostatic regulation socially dependent, whereby social input informs anticipatory control (*102*–*107*), constraining deviations before they unfold and accelerating recovery (*108*). This aligns with recent neuroscience frameworks positioning allostasis as a core feature of brain function (*23*, *109*), emphasizing predictive rather than reactive control of physiological regulation (*107*, *108*). Social embedding into neural predictive regulation may explain the domain-general regulatory function of social proximity, providing a potential account for Social Physiology (*48*). Socially informed allostasis may represent one route through which social relationships shape physiological regulation, alongside system-specific mechanisms. While distinct biological and behavioral pathways likely govern regulation within individual physiological systems, they converge on a common function: optimizing energetic efficiency. Social proximity may influence multiple physiological systems through domain-general allostatic processes, with system-specific mechanisms implementing these effects in a context-dependent manner. Glucose regulation provides a particularly stringent test of this account. Glucose metabolism is metabolically costly despite not necessarily being subjectively experienced or accompanied by a physiological stress response, as observed here. Yet, glucose regulatory efficiency was significantly improved by social proximity, suggesting that social influences may extend to homeostatic functions not traditionally considered socially regulated, potentially through predictive regulation (*54*).

The present findings establish Social Physiology as a proof of concept in close dyadic bonds, where social input is most likely to be physiologically relevant (*48*). They do not imply that all close bonds or social contexts uniformly enhance regulation nor that social proximity is invariably beneficial. Beyond replicable group-level effects, individual differences observed in this study reinforce this conclusion, as some participants exhibited substantial Social Physiological Gains with their partner, others showed modest effects, and a subset exhibited less efficient homeostasis in the Social condition (Social Physiological Loss). This opens an empirically testable avenue for examining how different social contexts shape the energetic costs of regulation.

While the present findings establish Social Physiology as a robust and replicable phenomenon, several limitations should be noted. First, the biological and behavioral pathways that facilitate Social Physiology warrant further investigation. Second, as the study is cross-sectional, it does not allow conclusions about the stability of these effects over time or their potential accumulation into long-term outcomes such as disease prevention and physiological resilience. In addition, the present research did not assess interactions among homeostatic systems despite the brain’s role in coordinating ongoing demands across multiple physiological systems. Relatedly, the observed effects could reflect interactions between social and metabolic demands, whereby engaging multiple challenges simultaneously alters the temporal dynamics of physiological responses. The presence of a social partner may also introduce additional environmental complexity that contributes to these effects. Such interactions may either facilitate regulation by promoting more efficient resolution of physiological challenges or impair regulation through competition for shared regulatory resources. Distinguishing between these possibilities remains an important direction for future research. Developmentally, only sympathetic regulation was assessed in infants. Given their dependence on caregiver regulation, effects may be stronger and extend across systems, warranting developmental and cross-system investigation. Last, cross-system effects were modest within participants and moderate between participants, consistent with our preregistered expectation that each experiment samples only a brief window of continuously operating regulation. Larger acute shifts would be unlikely in a system that must remain sustainable even in the absence of social proximity. However, even modest improvements, when sustained over time, may accumulate into a substantive energetic advantage of social proximity, potentially at a scale relevant for positive selection.

Together, these findings demonstrate that human physiology operates more efficiently in social proximity, indicating that homeostasis is not purely an individual process but can be socially enhanced. Social Physiology provides a biological framework for understanding the evolution of sociality and links between social connection and health.

## MATERIALS AND METHODS

The study comprised four experiments. The first was an exploratory Pilot Experiment, testing the concept of Social Physiology by examining EDA responses during a stress challenge in infants. On the basis of this Pilot Experiment, Experiments 1 to 3 were preregistered to systematically test Social Physiology in adults across three homeostatic systems: glucose metabolism during a carbohydrate challenge, thermoregulation during a cold challenge, and EDA response during a stress challenge. The study was preregistered on 9 April 2024 at https://osf.io/ before the adult data collection. We used ChatGPT to assist with R scripting and English language editing.

### Participants

#### Infants: Pilot Experiment

Seventy-two mothers (ages 22 to 47 years; mean = 31.73; SD = 5.08) and their infants (ages 2.5 to 13 months; mean = 6.51; SD = 2.8) were recruited to the study. Fourteen dyads were excluded because of incomplete measurements resulting in data loss, and two additional dyads were excluded because the Alone condition could not be completed as the infants became too distressed to continue. The final sample consisted of 56 mothers (age range: 22 to 47 years; mean = 32.04; SD = 5.26) and their 56 infants (27 females and 29 males; age range: 2.5 to 13 months; mean = 6.43; SD = 2.87).

#### Adults: Experiments 1 to 3

On the basis of the results of the Pilot Experiment, the sample size was determined using power analysis [G*Power (*110*)], specifying a mild to weak effect size and a power of 0.9 in a paired-sample *t* test. This conservative criterion was selected to minimize the risk of type II error. The target sample size was 120 participants for each experiment, recruited and tested as 60 romantic couples.

The adult studies included 180 unique participants (ages 18 to 37 years; mean = 24.81; SD = 2.36; 90 women). Each homeostatic system was tested in a separate experiment; thus, comparisons across systems were not conducted. Instead, each experiment served as a conceptual replication to evaluate the generalizability of Social Physiology across regulatory domains. Participation in one experiment did not preclude involvement in others, and some individuals took part in multiple experiments. In total, 339 adult experimental observations were completed across the three experiments (glucose: *n* = 108; temperature: *n* = 116; stress: *n* = 115). The distribution of unique participants was as follows: glucose only (*n* = 48 adults; 24 women), temperature only (*n* = 13; six women), stress only (*n* = 3; two women), glucose and temperature (*n* = 4; two women), glucose and stress (*n* = 13; six women), temperature and stress (*n* = 56; 28 women), and all three (*n* = 43; 22 women). To account for nonindependence from individuals contributing data to multiple experiments and from participants within the same dyad, analyses aggregating across experiments used multilevel linear models with random intercepts for participants nested within dyads. Participants were part of heterosexual couples in monogamous relationships of at least 9-month duration (*111*) and were tested with their partner.

In the Glucose Experiment (Experiment 1), of the 120 participants, six couples were excluded because they did not complete the full protocol (five couples did not successfully comply with the protocol requiring consumption of 10 g of carbohydrates before the experiment, and one couple could not complete the Social condition because of a technical equipment malfunction). Thus, analyses were conducted on 108 observations (108 unique participants; 54 romantic couples).

In the Thermoregulation Experiment (Experiment 2), of the 120 participants, four participants were excluded because they did not complete the protocol (one couple remained in the cold room for 7 min instead of the prescribed 5 min, the sensor of one participant malfunctioned during the experiment, and one participant wore a sweatshirt in the cold room, which was contrary to the lightweight clothing protocol). Thus, analyses were conducted on 116 observations (116 unique participants; 57 couples, one man, and one woman).

In the Stress Experiment (Experiment 3), of the 120 participants, two couples were excluded because of BIOPAC stimulator malfunction, and one participant was excluded because of a malfunction in electrode calibration. In total, analyses were conducted on 115 observations (115 unique participants; 57 couples and one woman).

Participants were recruited via social media platforms and the Hebrew University online experiment system. Inclusion criteria were assessed during a recruitment interview to confirm the absence of diabetes, head trauma, seizures, neurological or psychiatric disorders, and a body mass index score above 18 and below 30. Moreover, participants completed sociodemographic and health questionnaires. In the Glucose Experiment, participants were tested for HbA1c levels to ensure that they were neither prediabetic nor diabetic, with a maximum inclusion threshold of ≤5.6 (*31*). The study received ethical approval from the Institutional Review Board of the Hebrew University of Jerusalem (approval number: IRB_2024_66), and the procedures were conducted under the relevant guidelines and regulations. Before participation, all participants signed an informed consent.

### Experimental procedures

All experiments were conducted in a dedicated behavioral physiology laboratory. The testing room was spacious and furnished in a comfortable style, including a sofa, carpet, and natural light. This arrangement was designed to support naturalistic behavior, allowing participants to interact freely or remain quietly present while undergoing the experimental challenges.

In each experiment, the physiological challenge was administered twice: once in an Alone condition, where participants were tested individually, and once in a Social condition, where participants were tested with their partner. Both conditions were conducted on the same day, and the order of conditions was counterbalanced and randomly assigned across participants. Detailed order effects analyses are provided in section S4.1.2. In the Glucose and Temperature Experiments, the challenge was administered simultaneously to both partners (e.g., consuming carbohydrates or entering the cold room at the same time). In the Stress Experiment, because of the use of a single BIOPAC stimulator, partners received the challenge (electrical stimulation) in alternating turns, with the order of administration randomized.

#### Experimental manipulation: Social condition (naturalistic close-partner interactions)

In the Social condition, participants completed each physiological challenge with a close partner (romantic partner in the adult experiments and the mother in the infant experiment) present in the room. To maximize ecological validity and permit multiple social channels to operate, interactions were naturalistic: Partners were free to talk and touch at their discretion. The experimenter minimized on-site presence and refrained from interacting beyond safety procedures and task delivery. All sessions were continuously video recorded to document behavior. Videos were used to quantify behavior and assess the contribution of touch to Social Physiology.

#### Glucose Experiment

The experiment used a standardized carbohydrate-meal test: Participants ate 102 g of white wheat bread, providing 50 g of available carbohydrates (*26*). Fifty grams is the conventional dose used in glycemic-index and standardized carbohydrate-meal protocols (*11*, *26*). Capillary plasma glucose was measured at the baseline (before eating) and at 15, 30, 45, 60, 90, and 120 min after ingestion (*11*) with finger-stick sampling and the Accu-Chek Instant glucometer (*112*), each condition lasting for 2 hours post–food intake.

Participants were instructed to abstain from physical activity, food, and caloric beverages for 12 hours before the experiment. Unsweetened water-based coffee and green tea were permitted up to 1.5 hours before the session, and water was always allowed. To standardize baseline glucose levels between conditions, 2 hours before the first carbohydrate-meal test, participants ingested a standard plain biscuit, providing 10 g of available carbohydrate to abolish prolonged-fasting effects while avoiding residual hyperglycemia. This is based on previous research showing that in nonobese healthy adults, plasma glucose and insulin levels after a 10-g oral glucose load return to the baseline within 60 min (*113*), confirming that no carryover persists at the start of the first condition. Both experimental conditions (Social and Alone) were conducted on the same day, with a minimum of 4 hours between conditions.

##### Preprocessing and glucose AUC quantification

To generate a postprandial glycemic curve across the challenge for each participant, spline interpolation with four degrees of freedom was applied to the blood glucose measurements collected over 120 min, resulting in 100,000 interpolated data points. This approach produces a smooth glucose curve, avoiding overfitting and aligning with expected glucose dynamics (*114*, *115*). Normalization between conditions was performed by calculating the hyperglycemic AUC_0–120_ (iAUC; mg/dl·min), defined as the area above a threshold of plasma glucose levels of 100 mg/dl. This threshold represents the upper limit of normal fasting plasma glucose according to the American Diabetes Association (*116*, *117*). Accordingly, values exceeding 100 mg/dl were considered elevated and reflective of the postprandial glucose response (*118*).

#### Temperature Experiment

The temperature challenge consisted of a cold challenge with three phases: a 5-min baseline period (room temperature set to 25°C), a 5-min cold room period (room temperature set to 12°C), and a 15-min recovery period (room temperature set to 25°C), resulting in 25 min per condition. Participants’ clothing was controlled and consisted of lightweight attire throughout the cold challenge, with an additional warm layer worn during the recovery period. This procedure was repeated twice for both the Alone and Social conditions, with the order of conditions counterbalanced.

Participants’ skin temperature was continuously monitored using the EmbracePlus peripheral temperature sensor, a wireless wearable wristband device equipped with a digital sensor that records peripheral skin temperature at a sampling rate of 1 Hz (*119*). To minimize data loss and reduce artifacts, each participant wore two wristbands (one on each hand), and their AUC value for each condition was calculated as the average of the AUC values obtained from both sensors. Fifteen minutes before the experiment, participants were exposed to a brief 3-min cold stimulus to standardize baseline temperature levels across conditions. To control for fluctuating room temperatures, the temperatures in the cold and recovery rooms were monitored every 5 min using an atmospheric thermometer. The room temperature at the baseline and recovery was set to 25°C and ranged from 22° to 30°C. The room temperature in the cold room was set to 12°C and ranged from 11° to 18°C. The fluctuations in room temperature did not affect the Social Physiological Function, and the partner’s presence did not materially alter the room temperature during the cold challenge. Detailed analyses are provided in section S4.3.

##### Preprocessing and temperature AUC quantification

The raw data were temporally aligned using UNIX time stamps (*120*) (seconds since 00:00:00 UTC on 1 January 1970). Normalization between conditions was performed by calculating the percent change in skin temperature relative to the mean temperature during the 10-s preceding entry into the cold room (iAUC; % change in °C·min). Given that the cold challenge induced a reduction in peripheral temperature, the temperature values fell below the normalized baseline, resulting in negative values of percent signal. Because the AUC function does not treat negative values as a meaningful area, values below the threshold were converted to absolute values, allowing the quantification of the magnitude and duration of the temperature drop.

#### Stress Experiment

##### Adult couples

The stress challenge consisted of a stress paradigm with two mild electric shocks (7 V and 10 ms), applied over a 1-min “shock period.” We used a fixed, standardized shock intensity across participants to ensure that differences in physiological responses reflect modulation by social context, rather than differences in reported sensitivity. To account for interparticipant variability, we normalize the signal as the percent signal change so that the individual differences represent responsivity, rather than the absolute pain threshold. The “shock period” was preceded by a cue, with shocks delivered at pseudorandom times within that minute. The shocks were delivered via Ag-AgCl electrodes placed on the forearm using a BIOPAC Systems stimulator (*121*). The sympathetic response to the shocks was measured with EDA, using EL507A BIOPAC electrodes, at a sampling frequency of 2000 Hz. Each condition began with a 1-min rest period, followed by a 1-min shock period, and a 3-min recovery period between conditions. Two initial weak shocks of 4.5 V were administered to the participants before the experiment to level the baseline EDA levels between the conditions. This procedure was repeated for both the Alone and Social conditions, with the order of conditions counterbalanced.

EDA refers to the continuous variation in the electrical characteristics of the skin. Eccrine sweat glands secrete varying amounts of sweat, depending on the degree of sympathetic activation, and as more sweat is being secreted, EDA increases (*122*, *123*). Typically, EDA is measured as skin conductance by applying a small, constant voltage to the skin. Skin conductance can be calculated by measuring the current flow through the electrodes, as the voltage is kept constant (*122*, *124*, *125*). EDA is controlled by the sympathetic nervous system and reflects the level of physiological arousal (*126*). EDA is a validated and standard measure in physiology that increases during stressful situations (*127*, *128*).

##### Preprocessing and EDA AUC quantification

The raw data were extracted from AcqKnowledge software (*129*), the data acquisition and analysis software of the BIOPAC system. Data were acquired at 2000 Hz and down-sampled to 80 Hz using a mean value transformation (*130*). An interquartile range method was applied to identify and remove disconnects (*131*). Missing data points were interpolated on the basis of surrounding values using linear interpolation (*131*, *132*). To eliminate high-frequency noise, we applied a Butterworth low-pass filter [cutoff frequency: 1 Hz (*130*); order: 2] (*133*) implemented via the “Signal” package in R (*130*). Normalization between the two conditions was performed by calculating the percent signal change in EDA levels (μS) relative to the EDA during the first second of the stress period. The incremental AUC_0–60_ (iAUC; % change in μS·s) was calculated to capture the stress response, which typically comprises an initial increase in EDA followed by a decline below the baseline. Because the AUC function integrates only values above *y* = 0, baseline values were shifted to 100%. This vertical shift ensured that both the positive and negative deviations from the baseline contributed to the calculated area.

##### Mother-infant dyads

In infants, the stress challenge used a stress paradigm with a 2-s aversive sound at a high intensity (82 dB). The sound consisted of a recording of a metal pipe falling and was selected on the basis of prior research demonstrating its efficacy in eliciting a physiological response in infants measured with electromyography (*134*). The infants’ sympathetic response to the sound was measured with EDA using Empatica E4 wristbands placed on the infant’s wrist, sampling at a frequency of 4 Hz. Each condition began with a 5-min rest period, followed by a 22-s stress period, composed of a 2-s sound stimulus with 10 s of monitoring before and after the stressor. The experiment was conducted in participants’ homes, and procedures were halted if the infant showed signs of discomfort. During the Social condition, infants and mothers were in close proximity and were free to interact naturally, including physical contact (e.g., being held or sitting on the mother’s lap), play, vocalizations, and mutual gaze. During the Alone condition, the mother remained in a separate room and was not visible or audible to the infant. This procedure was repeated for both the Alone and Social conditions, with the order of conditions counterbalanced.

##### Preprocessing and EDA AUC quantification

The Empatica wristbands contain an EDA sensor with a sampling frequency of 4 Hz (*135*). The obtained electrodermal raw data were downloaded from the CareLab Portal (*119*), the data acquisition and analysis portal of the Empatica system, and temporally aligned on the basis of UNIX time stamps. Normalization between the two conditions was performed by calculating the percent signal change in EDA levels (μS) relative to the EDA during the first second of the stress period. The incremental AUC_0–22_ (iAUC; % change in μS·s) was calculated to quantify the full stress response, which involves an initial rise followed by a dip below the baseline. To ensure that both phases were captured, baseline levels started at 100%.

#### Quantifying Social Physiology

We calculated the Social Physiological Function for each participant in each experiment. For this, incremental AUC values were computed separately for the Social and Alone conditions in each experiment using the “auc” function from the “MESS” package in R, which applies the trapezoidal rule to approximate the integral over the estimated values paired with their corresponding time points (*136*), using a lower threshold of *y* = 0.

We then contrasted the AUC values of each physiological system (*P*) between the Alone and Social conditions

$$\text{Social physiological Function}={\text{iAUC}}_{P\left(\text{Alone}\right)}-i{\text{AUC}}_{P\left(\text{Social}\right)}$$

Positive values of the Social Physiological Function indicate a Social Physiological Gain, reflecting a more adaptive physiological response, with a smaller deviation from the baseline and/or quicker recovery in the Social condition compared to the Alone condition. Conversely, negative values indicate a Social Physiological Loss, signifying a more effortful physiological response with a bigger deviation and/or longer recovery in the Social condition compared to the Alone condition.

### Statistical analyses

#### Within-participant Social Physiological Gain

In each experiment, a pairwise *t* test was performed to quantify the Social Physiological Function by comparing the within-participant AUC values in each condition (Alone versus Social).

#### Main effect of Social Physiology across homeostatic systems

A linear mixed-effects model was used to examine the effect of condition (Social versus Alone) on AUC scores, standardized within each experiment. To aggregate responses from glucose, temperature, and sympathetic arousal, each measured on different scales, we computed the iAUC for each system and *z*-normalized values within the system. This approach enabled a domain-general analysis while preserving system-level variability. Random intercepts were specified for participants nested within a dyad to account for the hierarchical structure of the data (using the “lmer” function from the “lmeTest” package in R). This structure accounts for the nonindependence of observations across different experiments contributed by the same unique participant who participated in multiple experiments, as well as for potential shared variance introduced by participants belonging to the same dyad, given that each dyad consisted of a romantic couple.

#### Between-participant Social Physiological Gain

To address potential order effects, we also tested whether the Social Physiology effect emerges in a between-participant analysis restricted to the first session, either the Social or Alone condition. While condition order was counterbalanced, this analysis eliminated potential carryover from repeated physiological challenges in the within-participant analyses. For this, an independent-sample Welch *t* test was conducted on participants’ AUC values from their first session, either in the Social or Alone condition.

The significance level for the *t* tests or linear mixed-effects models calculating the Social Physiological Gain was set at *P* < 0.05 (one-tailed), given the a priori preregistered hypothesis that AUC values would be lower in the Social condition compared to the Alone condition. In the exploratory analyses of the supplementary sections, the significance level was set at *P* < 0.05 (two-tailed). All analyses were performed using R (version 4.2.2). Data are presented as the means ± SEM. The statistical effect size, significance values, and sample sizes are described in the figure legends.

## Supplementary Materials

### This PDF file includes:

Sections S1 to S4 Fig. S1 Table S1 References
