# Supplementary material for: Social Physiology: Human homeostasis is more efficient in social proximity
Source: Sci Adv. 2026 Sep 23;12(39):eaeg4937. doi: 10.1126/sciadv.aeg4937 (PMC13600219; doi:10.1126/sciadv.aeg4937)
Supplement: Supplementary file 1 [file sciadv.aeg4937_sm.pdf]

Science Advances
AAAS

Supplementary Materials for

Social Physiology: Human homeostasis is more efficient in social proximity

Monia Masalha *et al.*

Corresponding author: Shir Atzil, shir.atzil@mail.huji.ac.il

Sci. Adv. **12**, eaeg4937 (2026)
DOI: 10.1126/sciadv.aeg4937

This PDF file includes:

Sections S1 to S4
Fig. S1
Table S1
References

Supplementary Section 1: Within-Participant, Perturbation–Recovery, and Robustness Analyses of the Social Physiology Effect

<u>1.1. Homeostatic regulation is more efficient in a social context across physiological systems and age groups in within-participant analyses (Figure S1).</u>

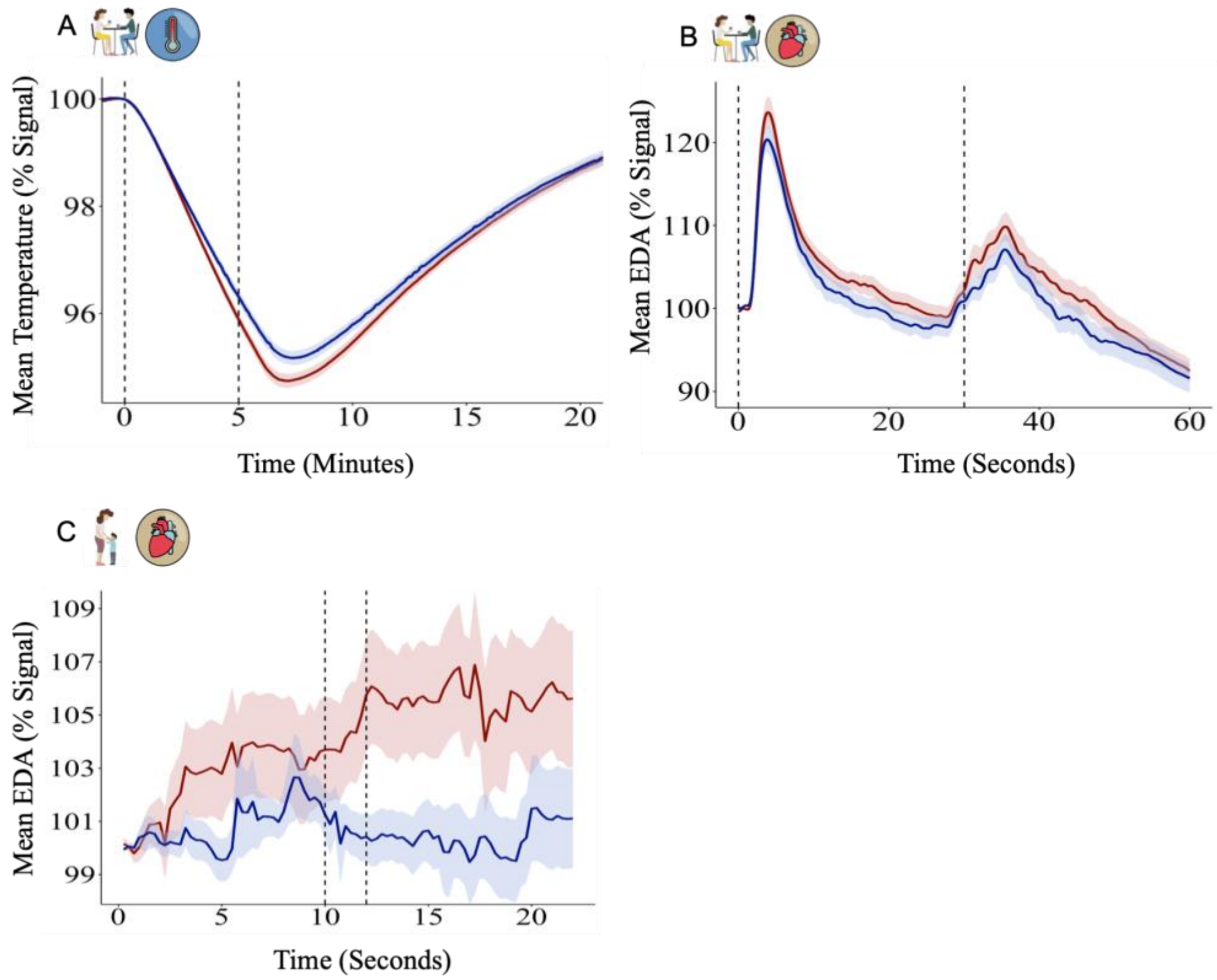

Figure S1. Homeostatic regulation is more efficient in a social context across physiological systems and age groups in within-participant analyses. Across systems and age groups, homeostatic regulation is more efficient in the Social condition (blue) than in the Alone condition (red), reflected in reduced perturbation amplitude and faster recovery. (**A**) The thermoregulatory response to the cold challenge is milder in the Social condition than in the Alone condition (within-participant analysis: $d = 0.17$; $p = 0.03$; $n = 116$). Dashed lines indicate cold-room entry and exit. Curves represent group means ± SEM. (**B**) The sympathetic response to an electric-shock is lower in the Social condition than in the Alone condition (within-participant analysis: $d = 0.13$; $p = 0.09$; $n = 115$). Dashed lines indicate shock administration. Curves represent group means ± SEM. (**C**) The sympathetic response to the aversive sound in infants is lower in the Social condition than in the Alone condition (within-participant analysis: $d = 0.23$; $p = 0.04$; $n = 56$). Dashed lines indicate aversive sound onset and offset. Curves represent group means ± SEM. Graphic illustrations were created by Y. Yadegari from HandMed.

1.2. Social proximity attenuates physiological perturbations and accelerates recovery

We tested the effect of social proximity on perturbation amplitude (peak deviation from baseline) and recovery dynamics (latency to onset of recovery). Across the Glucose and Temperature experiments, perturbation amplitude was significantly reduced and recovery latency was significantly shorter in the Social condition (Perturbation amplitude: Glucose Experiment: within-participant analysis: $t(107) = 3.16$; $d = 0.30$; $p = 0.001$; between-participant analysis: Welch $t(105) = 1.87$; $d = 0.36$; $p = 0.03$; Temperature Experiment: within-participant analysis: $t(115) = 3.29$; $d = 0.31$; $p = 0.007$; between-participant analysis: Welch $t(109) = 2.69$; $d = 0.26$; $p = 0.004$; Recovery latency: Glucose Experiment: within-participant analysis: $t(62) = 1.92$; $d = 0.24$; $p = 0.03$; between-participant analysis: Welch $t(82.5) = 2.01$; $d = 0.43$; $p = 0.02$; Temperature Experiment: within-participant analysis: $t(73) = 3.40$; $d = 0.40$; $p < 0.001$; between-participant analysis: Welch $t(104) = 2.10$; $d = 0.41$; $p = 0.02$).

For the Glucose Experiment, an additional threshold-based mixed-effects logistic analysis confirmed that social proximity accelerated return to normoglycemic levels of 110 (*31*), with sustained clearance. At 60 minutes postprandial, participants in the Social condition exhibited more than a twofold increase in the likelihood of returning to normoglycemic levels (odds ratio = 2.16, $p = 0.03$). This acceleration was consistently observed within participants across the recovery window (T_{90}: odds ratio = 1.77; $p = 0.04$; T_{120}: odds ratio = 1.95; $p = 0.02$).

In the Stress Experiment, neither perturbation amplitude nor recovery latency differed significantly between conditions (Perturbation amplitude: $t(114) = 0.38$; $p = 0.35$; Recovery latency: $t(108) = -1.74$; $p = 0.96$), consistent with the smaller effect size observed in this paradigm.

1.3. The Social Physiology effect is not driven by outliers

We tested the extent to which the social physiology effect is driven by outliers. Outliers exceeding ±3 standard deviations from the group mean were identified in 4 of 678 observations: two occurred in the Glucose Experiment (one in the Alone condition, and one in the Social condition) and two in the Stress Experiment (one in the Alone condition, and one in the Social condition). No extreme values were detected in the Temperature Experiment. The Social Physiology effect remained robust following their exclusion across systems and in the Glucose Experiment, and marginally

significant in the Stress Experiment (across homeostatic systems: $\beta = 0.11$; $p = 0.004$ (one-tailed $p = 0.002$); $n = 335$ paired observation (674 data points); Glucose Experiment: within-participant analysis: t(105) = 3.13; $d = 0.30$; $p = 0.001$; $n = 106$; between-participant analysis: Welch t(100.8) = 2.21; $d = 0.42$; $p = 0.015$; Stress Experiment: within-participant analysis: t(112) = 1.10; $p = 0.14$; $n = 113$; between-participant analysis: Welch t(105.1) = 2.59; $d = 0.49$; $p = 0.006$).

1.4. Relationship satisfaction did not account for the Social Physiology effect

We tested whether self-report of relationship satisfaction, assessed using the self-report Dyadic Adjustment Scale (DAS) (*137*) correlates with Social Physiology.

1.4.1. Relationship satisfaction did not correlate with AUC

We found no credible evidence for an association between DAS scores and AUC in neither condition nor system (Spearman correlation between participants' DAS scores and glucose AUC in the Alone condition: $r = -0.13$; $p = 0.20$; 95% *CI* [-0.31, 0.06]; BF01 = 2.05; $n = 108$; Spearman correlation between participants' DAS scores and glucose AUC in the Social condition: $r = 0.05$; $p = 0.62$; 95% *CI* [-0.14, 0.24]; BF01 = 4.02; $n = 108$; Spearman correlation between participants' DAS scores and temperature AUC in the Alone condition: $r = 0.10$; $p = 0.29$; 95% *CI* [-0.09, 0.28]; BF01 = 2.75; $n = 115$; Spearman correlation between participants' DAS scores and temperature AUC in the Social condition: $r = 0.11$; $p = 0.25$; 95% *CI* [-0.08, 0.29]; BF01 = 2.46; $n = 115$; DAS scores were not available for 1 out of 116 participants in the Temperature Experiment; Spearman correlation between participants' DAS scores and stress AUC in the Alone condition: $r = -0.05$; $p = 0.57$; 95% *CI* [-0.23, 0.12]; BF01 = 3.96; $n = 114$; Spearman correlation between participants' DAS scores and stress AUC in the Social condition: $r = 0.03$; $p = 0.79$; 95% *CI* [-0.16, 0.21]; BF01 = 4.48; $n = 114$; DAS scores were not available for 1 out of 115 participants in the Stress Experiment).

1.4.2. Relationship satisfaction levels did not mediate the Social Physiology effects

Controlling for relationship satisfaction in a linear mixed-effects model, the Social Physiology effect remained significant (across homeostatic systems: $\beta = 0.11$; $p = 0.002$ (one-tailed $p = 0.001$); 95% *CI* [0.04, 0.18]; $n = 337$; Glucose Experiment: $\beta = 0.14$; $p = 0.002$ (one-tailed $p = 0.001$);

95% *CI* [0.05, 0.24]; *n* = 108; Temperature Experiment: $\beta = 0.11$; $p = 0.07$ (one-tailed $p = 0.03$); 95% *CI* [-0.01, 0.24]; *n* = 115; Stress Experiment: $\beta = 0.08$; $p = 0.18$ (one-tailed $p = 0.09$); 95% *CI* [-0.04, 0.19]; *n* = 114).

1.5. Infant Age Does Not Account for the Social Physiology Effect

Infants' age range was between 2.5-13 months with Mean = 6.52; SD = 2.81; *n* = 55; age was not available for 1 out of 56 infants.

1.5.1. Infants' age did not correlate with EDA AUC

We found no credible evidence for an association between infants' age and sympathetic arousal AUC values in both conditions (Spearman correlation between infants age and sympathetic arousal AUC values in the Alone condition: $r = -0.006$; $p = 0.96$; 95% *CI* [-0.27, 0.26]; BF01 = 1.47; *n* = 55; age was not available for 1 out of 56 infants; Spearman correlation between infants age and sympathetic arousal AUC values in the Social condition: $r = -0.177$; $p = 0.20$; 95% *CI* [-0.42, 0.09]; BF01 = 1.23; *n* = 55). Moreover, we found no credible evidence for an association between infants' age and Social Physiological Gain (Spearman correlation between age and Social Physiological Gain scores: $r = 0.06$; $p = 0.69$; 95% *CI* [-0.21, 0.32]; BF01 = 0.78; *n* = 55; age was not available for 1 out of 56 infants).

Controlling for infants' age in a linear mixed-effects model, the Social Physiology effect remained evident, however marginally significant ($\beta = 36.63$; $p = 0.09$ (one-tailed $p = 0.045$); 95% *CI* [-5.82, 79.08]; *n* = 55).

Supplementary Section 2: Social Physiology is independent from physiological stress

2.1. Social Physiology persists after controlling for EDA

To assess the extent to which Social Physiological Gains reflect reduced stress in the presence of a familiar partner, we tested whether stress mediated the observed effects. Stress was assessed using electrodermal activity (EDA), a validated physiological marker of sympathetic arousal commonly used in stress research (*29*), recorded continuously during the Glucose Experiment with

a Biopac system and the Temperature Experiment with an Empatica ambulatory device (see Materials & Methods Section). For each participant, mean EDA levels were calculated separately for the baseline and challenges phases. In the Glucose Experiment, baseline was defined as the first five minutes of the experiment, starting prior to food intake, and the challenge phase spanned the 120-minute postprandial challenge period. In the Temperature Experiment, baseline was five minutes at room temperature, and the challenge phase was five minutes in the cold room. EDA was not tested as a mediator in the Stress Experiment, where it served as the primary outcome measure.

We tested whether EDA levels were elevated in the Alone condition compared to the Social condition. We then examined the association between EDA levels and iAUC values within each condition of each experiment using Spearman rank-order correlations. Moreover, we assessed the extent to which EDA contributed to the social physiology effect by re-estimating the primary models while statistically controlling for EDA levels using a linear mixed-effects model. The Social Physiology effect remained significant after controlling for EDA. For nonsignificant effects, we computed Bayes factors to assess evidence for the null versus alternative hypothesis. A Bayes Factor 01 (BF_{01}) between 1 and 3 suggests weak support for the null hypothesis, values between 3 and 10 indicate moderate support, and values greater than 10 provide strong evidence in favor of the null hypothesis (*138*).

Because electrodermal activity relies on sweat gland activity, cold temperatures may attenuate skin conductance responses and reduce the absolute magnitude of the EDA signal. To determine whether this affected the reliability of EDA measurement as a covariate in the cold challenge, we assessed the consistency of individual differences in EDA between the signal measured at room temperature and the signal measured during the cold challenge. EDA responses in the Temperature Experiment were lower in absolute magnitude than those observed in the Glucose Experiment (mean = 1.2 µS vs. 8.3 µS). However, despite the lower absolute values, EDA responses in the Temperature Experiment retained substantial variability and showed a strong within-participant correlation between room-temperature baseline and cold-challenge measurements, indicating that individual differences were preserved despite the cold environment and supporting its use as a covariate. Moreover, these differences should be interpreted cautiously, as the Temperature Experiment was conducted using Empatica devices, whereas the Glucose Experiment used Biopac systems, which may also affect absolute signal magnitude. Nonetheless, because cold exposure

may attenuate skin conductance responses, findings involving EDA in the Temperature Experiment should be interpreted with appropriate caution (Temperature: EDA in Alone condition; mean = 1.17 μS, SE = 0.17, range = 0.01–11.06 μS; EDA in Social condition; mean = 1.20 μS, SE = 0.17, range = 0.02–10.60 μS; Glucose: EDA in Alone condition; mean = 8.28 μS, SE = 0.41, range = 0.67–21.03 μS; EDA in Social condition; mean = 8.80 μS, SE = 0.43, range = 0.68–22.41 μS; Pearson correlation between EDA during baseline and during the cold challenge: Alone condition: $r = 0.97$, $p < 0.001$; Social condition: $r = 0.96$, $p < 0.001$; $n = 115$).

2.1.1. EDA levels were not elevated in the Alone condition

Baseline: We found no credible evidence for a difference in the mean EDA levels between the Alone and Social conditions during the baseline phase of the Glucose and Temperature experiments (Glucose Experiment: Alone condition: Mean = 9.39 μS; SD = 3.77; Social condition: Mean = 9.21 μS; SD = 4.08; paired-samples t(103) = 0.49; $p = 0.63$; BF01 = 8.20; $n = 104$; EDA data during baseline was not available for 4 out of 108 participants of the Glucose Experiment; Temperature Experiment: Alone condition: Mean = 1.45 μS; SD = 2.15; Social condition: Mean = 1.52 μS; SD = 2.35; paired-samples t(114) = -0.56; $p = 0.57$; BF01 = 8.27; $n = 115$; EDA data during baseline was not available for 1 out of 116 participants of the Temperature Experiment).

Challenge: We found no credible evidence for a difference in mean EDA levels between the Alone and Social conditions during the challenge phase of the Glucose and Temperature experiments (Glucose Experiment, 120-minute post-prandial window: Alone condition: Mean = 8.28 μS; SD = 3.62; Social condition: Mean = 8.80 μS; SD = 4.20; paired-samples t(103) = -1.52; $p = 0.13$; BF01 = 3.02; $n = 104$; EDA data during challenge was not available for 4 out of 108 participants of the Glucose Experiment; Temperature Experiment, 5-minute of cold room: Alone condition: Mean = 1.17 μS; SD = 1.87; Social condition: Mean = 1.20 μS; SD = 1.84; paired-samples t(115) = -0.38; $p = 0.71$; BF01 = 9.06; $n = 116$).

2.1.2. EDA levels did not correlate with glucose or temperature AUC

We found no credible evidence for an association between EDA levels during the Glucose and Temperature experiments and the glucose or temperature AUC values in either condition (Glucose Experiment: Spearman correlation between EDA during the post-prandial glycemic challenge in the Alone condition and glucose AUC in the Alone condition: $r = -0.02$; $p = 0.81$; 95% *CI* [-0.23, 0.17]; BF01 = 4.31; $n = 104$; Spearman correlation between EDA during the post-prandial

glycemic challenge in the Social condition and glucose AUC in the Social condition: $r = 0.07$; $p = 0.45$; 95% *CI* [-0.12, 0.27]; BF01 = 3.40; $n = 104$; Temperature Experiment: Spearman correlation between EDA during the cold challenge in the Alone condition and temperature AUC in the Alone condition: $r = -0.04$; $p = 0.67$; 95% *CI* [-0.23, 0.15]; BF01 = 4.28; $n = 116$; Spearman correlation between EDA during the cold challenge in the Social condition and temperature AUC in the Social condition: $r = 0.11$; $p = 0.26$; 95% *CI* [-0.08, 0.28]; BF01 = 2.56; $n = 116$).

2.1.3. EDA levels did not mediate the Social Physiology effects in glucose and temperature regulation

Controlling for EDA levels in a linear mixed-effects model, the Social Physiology effect remained significant (nested within participants; Glucose Experiment: $\beta = 0.15$; $p = 0.002$ (one-tailed $p = 0.001$); 95% *CI* = [0.05, 0.24]; $n = 104$; Temperature Experiment: $\beta = 0.11$; $p = 0.07$ (one-tailed $p = 0.03$); 95% *CI* = [-0.01, 0.23]; $n = 116$).

Supplementary Section 3: The Social Physiology Effect Persists After Controlling for Physical Touch

We examined whether social touch, through which bodies directly influence one another, accounted for the observed Social Physiological Gains. All experimental sessions were video-recorded to allow systematic quantification of touch during the challenge tasks. Touch was coded second by second by trained coders, and inter-rater reliability was assessed on at least 10% of the recordings, yielding an agreement rate exceeding 90%. For each participant, touch was quantified as the percentage of total duration during which physical contact occurred.

Touch was coded across multiple body locations (head/neck, lips, torso, and extremities) and aggregated to yield a total bodily touch measure for each participant. In the Glucose Experiment, which lasted two hours, touch was coded across three one-minute segments (i.e., the first minute of food consumption; the first minute immediately following consumption; and the fifth minute post-consumption). Repeated segments enabled reliable quantification of behavior across the task. In the Temperature Experiment, touch was coded continuously throughout the five-minute cold exposure. In the Stress Experiment, touch was coded during the one-minute stress period;

3.1. Touch was not associated with AUC in the Glucose and Stress experiments but was associated with thermoregulation AUC

We found no credible evidence for an association between physical touch and Social AUC in the Glucose and Stress Experiments (Glucose Experiment: Spearman correlation between physical touch and glucose AUC in the Social condition: $r = -0.08$; $p = 0.45$; BF01 = 3.38; $n = 104$; physical touch data was not available for 4 out of 108 participants of the Glucose Experiment; Stress Experiment: Spearman correlation between physical touch and stress AUC in the Social condition: $r = 0.04$; $p = 0.69$; BF01 = 4.27; $n = 113$; physical touch data was not available for 2 out of 115 participants of the Stress Experiment). However, we found a negative association between physical touch and Social AUC in the Temperature Experiment (Spearman correlation between physical touch and temperature AUC in the Social condition: $r = -0.19$; $d = -0.39$; $p = 0.04$; $n = 113$; physical touch data was not available for 3 out of 116 participants of the Temperature Experiment). Moreover, beyond overall touch, touch duration of specific body regions, including the torso, was not reliably correlated with temperature AUC after adjusting for multiple testing (Table S1).

3.2. Touch did not mediate the Social Physiology effects in glucose metabolism

Controlling for touch levels in a linear mixed-effects model, the Social Physiology effect remained significant across homeostatic systems and specifically in the Glucose Experiment (across homeostatic systems: $\beta = 0.10$; $p = 0.005$ (one-tailed $p = 0.002$); 95% *CI* [0.03, 0.18]; $n = 330$; Glucose Experiment: $\beta = 0.14$; $p = 0.004$ (one-tailed $p = 0.002$); 95% *CI* [0.05, 0.23]; $n = 104$). In the Temperature Experiment, the effect remained significant only under the preregistered one-tailed test ($\beta = 0.10$; $p = 0.09$ (one-tailed $p = 0.047$); 95% *CI* [-0.02, 0.23]; $n = 113$). In the Stress Experiment, the effect remained not significant ($\beta = 0.07$; $p = 0.23$ (one-tailed $p = 0.11$); 95% *CI* [-0.04, 0.18]; $n = 113$).

Taken together, these findings suggest that physical touch may contribute to thermoregulation but does not provide a consistent account of the observed Social Physiology effects. Given the exploratory nature of the touch analyses, future research should determine whether, and under what contextual or bodily conditions, touch contributes to Social Physiology.

Supplementary Section 4: Controlling for Methodological Confounds

We tested the robustness of the Social Physiology effect by accounting for methodological confounds that could potentially influence the amplitude or duration of the physiological response. These included condition order, experiment start time, and Body Mass Index (BMI) (across all experiments), eating duration and Hemoglobin A1C (HbA1C) levels (in the Glucose experiment); room temperature fluctuations (in the Temperature experiment).

We examined the extent to which each confound was associated with iAUC values in each condition using Spearman rank-order correlations. Moreover, we assessed the extent to which each confound contributed to the Social Physiology effect by re-estimating the primary models while statistically controlling for each confound using a linear mixed-effects model. The Social Physiology effect remained significant after controlling for all confounds.

4.1. Controlling for methodological confounds relevant to all experiments

4.1.1. Experiment time did not influence the Social Physiology effect

The starting time varied across experiments. In the Glucose Experiment, sessions began between 9:00 AM and 1:00 PM; in the Temperature Experiment, start times ranged from 9:00 AM to 6:00 PM; and in the Stress Experiment, sessions were conducted between 10:00 AM and 8:00 PM.

4.1.1.1. Experiment starting time did not correlate with AUC values

We found no credible evidence for an association between experiment starting time and AUC values in the Glucose, Temperature or Stress experiments (Glucose Experiment: Spearman correlation between Glucose Experiment starting time and glucose AUC values in the Alone condition: $r = 0.16$; $p = 0.11$; 95% *CI* [-0.03, 0.34]; BF01 = 1.29; $n = 108$; Spearman correlation between Glucose Experiment starting time and glucose AUC values in the Social condition: $r = 0.11$; $p = 0.25$; 95% *CI* [-0.08, 0.31]; BF01 = 2.38; $n = 108$; Temperature Experiment: Spearman correlation between Temperature Experiment starting time and temperature AUC values in the Alone condition: $r = 0.07$; $p = 0.47$; 95% *CI* [-0.11, 0.25]; BF01 = 3.65; $n = 116$; Spearman correlation between Temperature Experiment starting time and temperature AUC values in the Social condition: $r = 0.02$; $p = 0.85$; 95% *CI* [-0.17, 0.21]; BF01 = 4.59; $n = 116$; Stress

Experiment: Spearman correlation between Stress Experiment starting time and stress AUC values in the Alone condition: *r* = 0.003; *p* = 0.97; 95% *CI* [-0.19, 0.20]; BF01 = 4.61; *n* = 113; the experiment start time was not available for 2 out of 115 participants in the Stress Experiment; Spearman correlation between Stress Experiment starting time and stress AUC values in the Social condition: *r* = 0.09; *p* = 0.32; 95% *CI* [-0.09, 0.27]; BF01 = 2.90; *n* = 113; the experiment start time was not available for 2 out of 115 participants in the Stress Experiment).

4.1.1.2. Experiment starting time did not mediate the Social Physiology effect

Controlling for experiment starting time in a linear mixed-effects model, the Social Physiology effect remained significant across homeostatic systems and in the Glucose and Temperature experiments and marginally significant in the Stress Experiment (across homeostatic systems: β = 0.11; *p* = 0.002 (one-tailed *p* = 0.001); 95% *CI* [0.04, 0.18]; *n* = 337; Glucose Experiment: β = 0.14; *p* = 0.002 (one-tailed *p* = 0.001); 95% *CI* [0.05, 0.24]; *n* = 108; Temperature Experiment: β = 0.11; *p* = 0.07 (one-tailed *p* = 0.03); 95% *CI* [-0.01, 0.23]; *n* = 116; Stress Experiment: β = 0.08; *p* = 0.17 (one-tailed *p* = 0.08); 95% *CI* [-0.03, 0.19]); *n* = 113).

4.1.2. The Social Physiology effect remains significant after controlling for condition order

Since both conditions were administered on the same day, an order effect on physiological responses was anticipated. To account for this, the order of conditions was counterbalanced and randomized across participants in each experiment. In addition to the experimental control, we also quantified the magnitude of the order effect within each physiological system and assessed its effects on the Social Physiological Gain.

We compared AUC values between first and second administrations using paired-samples t-tests to assess potential spillover effects on physiological responses, irrespective of whether the initial condition was Alone or Social. We found evidence for a significant order effect in the Glucose and Temperature experiments, potentially reflecting increased AUC values in the second condition (Glucose Experiment: First challenge: Mean = 1593.83; SD = 1037.88; Second challenge: Mean = 1909.32; SD = 1348.04; t(107) = -2.77; *d* = 0.27; *p* = 0.01; *n* = 108; Temperature Experiment: First challenge: Mean = 3269.38; SD = 1020.16; Second challenge: Mean = 3903.55; SD = 1134.39; t(115) = -5.02; *d* = 0.47; *p* < 0.001; *n* = 116). In the Stress Experiment, we found no evidence for order effect (Stress Experiment: First challenge: Mean = 6192.57; SD = 943.23; Second challenge: Mean = 6047.53; SD = 656.93; t(114) = 1.60; *p* = 0.11; BF01 = 2.81; *n* = 115).

4.1.2.1. Condition order did not mediate the Social Physiology effect

Controlling for order of conditions in linear mixed-effects model, with condition order included as a covariate, the Social Physiology effect remained significant across homeostatic systems and in the Glucose and Temperature experiments, and marginally significant in the Stress Experiment (across homeostatic systems: $\beta = 0.11$; $p = 0.002$ (one-tailed $p = 0.001$); 95% *CI* [0.04, 0.18]; $n = 339$; Glucose Experiment: $\beta = 0.14$; $p = 0.002$ (one-tailed $p = 0.001$); 95% *CI* [0.06, 0.23]; $n = 108$; Temperature Experiment: $\beta = 0.11$; $p = 0.04$ (one-tailed $p = 0.02$); 95% *CI* [0.003, 0.222]; $n = 116$; Stress Experiment: $\beta = 0.08$; $p = 0.18$ (one-tailed $p = 0.09$); 95% *CI* [-0.03, 0.18]; $n = 115$).

4.1.3. Participants' BMI did not influence the Social Physiology effect

4.1.3.1. BMI was associated with AUC only in the Alone condition of the Glucose Experiment

We found a significant association between participants' BMI and AUC values in the Alone condition of the Glucose Experiment, such that lower BMI scores were associated with improved glucose metabolism in the Alone condition (Spearman correlation between participants' BMI and glucose AUC in the Alone condition: $r = -0.27$; $p < 0.01$; 95% *CI* [-0.43, -0.09]; $n = 108$). We did not find evidence for a significant association between participants' BMI and AUC values in the Social condition of the Glucose Experiment, nor in any condition of the Temperature and Stress experiments (Glucose Experiment: Spearman correlation between participants' BMI and glucose AUC in the Social condition: $r = -0.14$; $p = 0.15$; 95% *CI* [-0.33, 0.05]; BF01 = 1.68; $n = 108$; Temperature Experiment: Spearman correlation between participants' BMI and temperature AUC in the Alone condition: $r = -0.15$; $p = 0.12$; 95% *CI* [-0.33, 0.05]; BF01 = 1.43; $n = 115$; Spearman correlation between participants' BMI and temperature AUC in the Social condition: $r = 0.05$; $p = 0.59$; 95% *CI* [-0.15, 0.24]; BF01 = 4.04; $n = 115$; BMI was not available for 1 out of 116 participants in the Temperature Experiment; Stress Experiment: Spearman correlation between participants' BMI and stress AUC in the Alone condition: $r = 0.04$; $p = 0.69$; 95% *CI* [-0.15, 0.22]; BF01 = 4.30; $n = 114$; Spearman correlation between participants' BMI and stress AUC in the Social condition: $r = 0.02$; $p = 0.80$; 95% *CI* [-0.17, 0.21]; BF01 = 4.49; $n =114$; BMI was not available for 1 out of 115 participants in the Stress Experiment).

4.1.3.2. Participants' BMI did not mediate the Social Physiology effect

Controlling for BMI scores in a linear mixed-effects model, the Social Physiology effect remained significant (across homeostatic systems: $\beta = 0.11$; $p = 0.002$ (one-tailed $p = 0.001$); 95% *CI* [0.04, 0.18]; $n = 337$; Glucose experiment: $\beta = 0.14$; $p = 0.002$ (one-tailed $p = 0.001$); 95% *CI* [0.05, 0.24]; $n = 108$; Temperature experiment: $\beta = 0.12$; $p = 0.05$ (one-tailed $p = 0.03$); 95% *CI* [0, 0.24]; $n = 115$; Stress experiment: $\beta = 0.08$; $p = 0.18$ (one-tailed $p = 0.09$); 95% *CI* [-0.04, 0.19]; $n = 114$).

4.2. Controlling for methodological confounds in the Glucose Experiment

4.2.1. Eating duration did not influence the Social Physiology effect

In the standardized carbohydrate meal test, participants were instructed to consume 50g of carbohydrates (102g of bread) within 5 minutes. However, actual eating durations varied and were individually quantified by identifying the start and end times of eating from video recordings of the session.

4.2.1.1. Eating duration correlated with glucose AUC

Eating duration was significantly longer in the Social condition than in the Alone condition (Alone condition: Mean = 4.63 minutes; SD = 1.85 minutes; Social condition: Mean = 6.04 minutes; SD = 2.63 minutes; paired-samples $t(100) = -5.26$; $d = -0.52$; $p < 0.001$; $n = 101$; video recordings were not available for 7 out of 108 participants, 3 participants in the Alone condition and 4 participants in the Social condition).

We found an association between eating duration and plasma glucose AUC only in the Alone condition (Spearman correlations between eating duration in the Alone condition and glucose AUC in the Alone condition: $r = 0.22$; $p = 0.03$; 95% *CI* = [0.02, 0.40]; $n = 105$; Spearman correlations between eating duration and glucose AUC in the Social condition: $r = -0.01$; $p = 0.93$; 95% *CI* = [-0.21, 0.19]; BF01 = 4.42; $n = 104$).

4.2.1.2. Eating duration did not mediate the glucose Social Physiology effect

Controlling for eating duration in a linear mixed-effects model, the Social Physiology effect remained significant ($\beta = 0.15$; $p = 0.003$ (one-tailed $p = 0.002$); 95% *CI* [0.05, 0.26]; $n = 104$).

4.2.2. HbA1C levels did not influence the Social Physiology effect

HbA1C reflects the average blood glucose levels over the past three months(*31*). HbA1C levels were measured for participants in the Glucose Experiment to verify that glucose regulation was within the normal range (*139*), and to screen for potential cases of diabetes or prediabetes (*139*). All participants fell within the normative range (range = 4.2% - 5.5%; Mean = 4.95; SD = 0.26; $n = 107$; HbA1C was not available for 1 out of 108 participants).

4.2.2.1. HbA1C did not correlate with glucose AUC

We found no credible evidence of an association between HbA1C levels and glucose AUC values in either condition (Spearman correlation between HbA1C levels and glucose AUC in Alone condition: $r = 0.01$; $p = 0.90$; 95% *CI* [-0.17, 0.20]; BF01 = 4.46; $n = 107$; Spearman correlation between HbA1C levels and glucose AUC in the Social condition: $r = 0.02$; $p = 0.86$; 95% *CI* [-0.17, 0.21]; BF01 = 4.43, $n = 107$).

4.2.2.2. HbA1C did not mediate the glucose Social Physiology effect

Controlling for HbA1C levels in a linear mixed-effects model, the effect of Social Physiology effect remained significant ($\beta = 0.15$; $p = 0.001$ (one-tailed $p < 0.001$); 95% *CI* [0.06, 0.24]; $n = 107$).

4.3. Controlling for methodological confounds in the Temperature Experiment

4.3.1. Fluctuations in room temperature did not influence the Social Physiology effect

The temperature during the cold challenge was set to 12°C, and in the recovery room, it was set to 25°C. To account for potential atmospheric fluctuations, the actual room temperature during the challenge and recovery periods was monitored at five-minute intervals using an atmospheric thermometer.

We found no credible evidence for a difference in room temperature fluctuations between the Social and Alone conditions (Cold challenge: Alone condition: Mean = 14.10°C; SD = 1.01°; Social condition: Mean = 14°C; SD = 1.09°; Welch t(228.08) = 0.70; $p = 0.45$; BF01 = 5.53; n = 115, room temperature data was not recorded in the Alone condition for 1 out of 116 participants during the cold challenge; Recovery: Alone condition: Mean = 26.10°C; SD = 1.39°; Social condition: Mean = 26.42°C; SD = 1.42°; Welch t(229.88) = -1.96; $p = 0.051$; BF01 = 1.15; n = 116).

<u>4.3.1.1. Fluctuations in room temperature did not correlate with temperature AUC values</u>

We found no credible evidence for an association between room temperatures and temperature AUC values in both conditions (Spearman correlation between cold room temperature in the Alone condition and temperature AUC in the Alone condition: $r = 0.01$; $p = 0.95$; 95% *CI* [-0.19, 0.20]; BF01 = 4.64; $n = 115$; room temperature was not recorded in the Alone condition for 1 out of 116 participants during the cold challenge; Spearman correlation between recovery room temperature in the Alone condition and temperature AUC in the Alone condition: $r = 0.10$; $p = 0.29$; 95% *CI* [-0.10, 0.29]; BF01 = 2.72; $n = 116$; Spearman correlation between cold room temperature in the Social condition and temperature AUC in the Social condition: $r = -0.11$; $p = 0.22$; 95% *CI* [-0.30, 0.08]; BF01 = 2.31; $n = 116$; Spearman correlation between recovery room temperature in the Social condition and temperature AUC in the Social condition: $r = 0.004$; $p = 0.97$; 95% *CI* [-0.18, 0.18]; BF01 = 4.67; $n = 116$).

<u>4.3.1.2. Fluctuations in room temperature did not mediate the temperature Social Physiology effect</u>

Controlling for the fluctuations in room temperature in a linear mixed-effects model, the Social Physiology effect remained significant ($\beta = 0.12$; $p = 0.05$ (one-tailed $p = 0.03$); 95% *CI* [0, 0.25]; $n = 115$).

<u>4.3.2. Social thermoregulation is more efficient in women than men</u>

An exploratory analysis revealed that the social thermoregulation effect was stronger in women (within-participant analysis: Females: Alone AUC: *Mean* = 3712.79 °C·min; *SE* = 96.86 °C·min; Social AUC: *Mean* = 3351.05 °C·min; *SE* = 175.15 °C·min; paired-samples $t(57) = 3.34$; $d = 0.44$; $p < 0.001$; a 16.00% mean reduction in the Social vs. Alone condition; $n = 58$; Males: Alone AUC: *Mean* = 3418.11°C·min; *SE* = 127.89°C·min; Social AUC: *Mean* = 3569.23°C·min; *SE* =

133.96°C·min; paired-samples $t(57) = -0.85$; $p = 0.78$; BF01 = 4.96; $n = 58$; Between-participant analysis: Females: Alone AUC: *Mean* = 3819.63°C·min; *SE* = 159.03 °C·min; $n_{(Alone)} = 29$; Social AUC: *Mean* = 2879.63°C·min; *SE* = 206.76 °C·min; $n_{(Social)} = 29$; Welch $t(52.54) = 3.60$; $d = 0.95$; $p < 0.001$; a 24.61% mean reduction in the Social vs. Alone conditions; Males: Alone AUC: *Mean* = 3294.28°C·min; *SE* = 185.83 °C·min; $n_{(Alone)} = 29$; Social AUC: *Mean* = 3083.96°C·min; *SE* = 165.32°C·min; $n_{(Social)} = 29$; Welch $t(55.25) = 0.85$; $p = 0.20$; BF01 = 2.79). Such sex differences were not evident in the Glucose and Stress experiments.

To examine whether these sex differences were driven by variations in touch frequency, we conducted exploratory sex-stratified analyses of touch across body regions (head & neck, lips, torso, extremities, and total) (Table S1). Among women, initial unadjusted correlations emerged between temperature AUC in the Social condition and touch at the head and neck ($r = -0.28$, $p = 0.033$), extremities ($r = -0.32$, $p = 0.016$), and total touch ($r = -0.29$, $p = 0.026$). No significant associations were observed among men. However, given the exploratory nature of these subgroup analyses and the number of statistical tests, none of these correlations survived Bonferroni correction. Consequently, these patterns should be interpreted with caution as preliminary and hypothesis-generating rather than confirmatory.

Table S1: Region-Specific Touch Correlations with Thermoregulation Overall and by Sex

Participants	Touch region	Correlation with temperature AUC during the social condition
All Participants (N = 113) corrected for 5 comparisons	Head & Neck	r= -0.10; p= 0.271; corrected p= 1
	Lips	r= -0.04; p= 0.694; corrected p= 1
	Torso	r= -0.07; p= 0.432; corrected p= 1
	Extremities	r= -0.24; p= 0.012; corrected p= 0.059
	Total	r= -0.19; p= 0.041; corrected p= 0.205
Males (N = 56) corrected for 10 comparisons	Head & Neck	r= 0.18; p= 0.191; corrected p= 1
	Lips	r= 0.11; p= 0.400; corrected p= 1
	Torso	r= 0.14; p= 0.313; corrected p= 1
	Extremities	r= -0.12; p= 0.379; corrected p= 1
	Total	r= -0.04; p= 0.749; corrected p= 1
Females (N = 57) corrected for 10 comparisons	Head & Neck	r= -0.28; p= 0.033; corrected p= 0.331
	Lips	r= -0.15; p= 0.274; corrected p= 1
	Torso	r= -0.23; p= 0.090; corrected p= 0.901
	Extremities	r= -0.32; p= 0.016; corrected p= 0.158
	Total	r= -0.29; p= 0.026; corrected p= 0.259

REFERENCES

1. J. E. Niven, S. B. Laughlin, Energy limitation as a selective pressure on the evolution of sensory systems. *J. Exp. Biol.* **211**, 1792–1804 (2008).

2. H. M. Dunsworth, A. G. Warrener, T. Deacon, P. T. Ellison, H. Pontzer, Metabolic hypothesis for human altriciality. *Proc. Natl. Acad. Sci. U.S.A.* **109**, 15212–15216 (2012).

3. P. Sterling, S. Laughlin, *Principles of Neural Design* (MIT Press, Cambridge, MA, USA, 2017).

4. H. Pontzer, Energy expenditure in humans and other primates: A new synthesis. *Ann. Rev. Anthropol.* **44**, 169–187 (2015).

5. D. Raubenheimer, S. J. Simpson, Nutritional ecology and human health. *Annu. Rev. Nutr.* **36**, 603–626 (2016).

6. J. E. Theriault, C. Shaffer, G. A. Dienel, C. Y. Sander, J. M. Hooker, B. C. Dickerson, L. F. Barrett, K. S. Quigley, A functional account of stimulation-based aerobic glycolysis and its role in interpreting BOLD signal intensity increases in neuroimaging experiments. *Neurosci. Biobehav. Rev.* **153**, 105373 (2023).

7. H. Pontzer, Y. Yamada, H. Sagayama, P. N. Ainslie, L. F. Andersen, L. J. Anderson, L. Arab, I. Baddou, K. Bedu-Addo, E. E. Blaak, S. Blanc, A. G. Bonomi, C. V. C. Bouten, P. Bovet, M. S. Buchowski, N. F. Butte, S. G. Camps, G. L. Close, J. A. Cooper, R. Cooper, S. K. Das, L. R. Dugas, U. Ekelund, S. Entringer, T. Forrester, B. W. Fudge, A. H. Goris, M. Gurven, C. Hambly, A. El Hamdouchi, M. B. Hoos, S. Hu, N. Joonas, A. M. Joosen, P. Katzmarzyk, K. P. Kempen, M. Kimura, W. E. Kraus, R. F. Kushner, E. V. Lambert, W. R. Leonard, N. Lessan, C. Martin, A. C. Medin, E. P. Meijer, J. C. Morehen, J. P. Morton, M. L. Neuhouser, T. A. Nicklas, R. M. Ojiambo, K. H. Pietiläinen, Y. P. Pitsiladis, J. Plange-Rhule, G. Plasqui, R. L. Prentice, R. A. Rabinovich, S. B. Racette, D. A. Raichlen, E. Ravussin, R. M. Reynolds, S. B. Roberts, A. J. Schuit, A. M. Sjödin, E. Stice, S. S. Urlacher, G. Valenti, L. M. Van Etten, E. A. Van Mil, J. C. K. Wells, G. Wilson, B. M. Wood, J. Yanovski, T. Yoshida, X. Zhang, A. J. Murphy-Alford, C. Loechl, A. H. Luke, J. Rood, D. A. Schoeller, K. R. Westerterp, W. W.

Wong, J. R. Speakman, IAEA DLW Database Consortium, Daily energy expenditure through the human life course. *Science* **373**, 808–812 (2021).

8. A. Behnke, E. Shaulson, H. Pontzer, C. P. Kempes, M. Picard, Energy constraint on human health. *Trends Endocrinol. Metab.* **S1043–2760**, 00045–7 (2026).

9. R. E. Lean, A. Constantino-Pettit, L. S. Gorham, M. P. Herzberg, B. Anaya, C. E. Rogers, J. L. Luby, Social determinants of health, the developing brain, and risk and resilience for psychopathology. *Neuropsychopharmacology* **51**, 185–202 (2026).

10. M. W. Schwartz, R. J. Seeley, M. H. Tschöp, S. C. Woods, G. J. Morton, M. G. Myers, D. D'Alessio, Cooperation between brain and islet in glucose homeostasis and diabetes. *Nature* **503**, 59–66 (2013).

11. D. J. Jenkins, T. M. Wolever, R. H. Taylor, H. Barker, H. Fielden, J. M. Baldwin, A. C. Bowling, H. C. Newman, A. L. Jenkins, D. V. Goff, Glycemic index of foods: A physiological basis for carbohydrate exchange. *Am. J. Clin. Nutr.* **34**, 362–366 (1981).

12. S. J. Cooper, From Claude Bernard to Walter Cannon. Emergence of the concept of homeostasis. *Appetite* **51**, 419–427 (2008).

13. D. F. Wilson, Programming and regulation of metabolic homeostasis. *Am. J. Physiol. Endocrinol. Metab.* **308**, E506–E517 (2015).

14. C. Trumpff, D. Shire, J. Michelson, N. Bobba-Alves, T. Yu, R. P. Sloan, R.-P. Juster, M. Hirano, M. Picard, Saliva and blood cell-free mtDNA reactivity to acute psychosocial stress. *Psychoneuroendocrinology* **179**, 107506 (2025).

15. N. Bobba-Alves, R.-P. Juster, M. Picard, The energetic cost of allostasis and allostatic load. *Psychoneuroendocrinology* **146**, 105951 (2022).

16. E. L. Rezende, L. D. Bacigalupe, Thermoregulation in endotherms: Physiological principles and ecological consequences. *J. Comp. Physiol. B* **185**, 709–727 (2015).

17. K. A. Ingraham, D. P. Ferris, C. D. Remy, Evaluating physiological signal salience for estimating metabolic energy cost from wearable sensors. *J. Appl. Physiol.* **126**, 717–729 (2019).

18. B. S. McEWEN, Stress, adaptation, and disease: Allostasis and allostatic load. *Ann. N. Y. Acad. Sci.* **840**, 33–44 (1998).

19. V. Eisner, M. Picard, G. Hajnóczky, Mitochondrial dynamics in adaptive and maladaptive cellular stress responses. *Nat. Cell Biol.* **20**, 755–765 (2018).

20. Q. Huang, C. Trumpff, A. S. Monzel, S. Rausser, J. Devine, C. C. Liu, C. Kelly, M. Kurade, S. Li, K. Engelstad, K. Tanji, V. Lauriola, T. Wang, S. Wang, R. Sloan, R.-P. Juster, M. Hirano, M. Picard, The mitochondrial disease biomarker GDF15 is dynamic, quantifiable in saliva, and correlates with disease severity. *Mol. Genet. Metab.* **145**, 109179 (2025).

21. M. Picard, R.-P. Juster, B. S. McEwen, Mitochondrial allostatic load puts the "gluc" back in glucocorticoids. *Nat. Rev. Endocrinol.* **10**, 303–310 (2014).

22. W. B. Cannon, Organization for physiological homeostasis. *Physiol. Rev.* **9**, 399–431 (1929).

23. M. Cohen, S. Atzil, A metabolic framework for reward: Redefining dopamine and opioids as physiological agents. *Neurosci. Biobehav. Rev.* **184**, 106608 (2026).

24. R. Peters, D. J. White, B. R. Cornwell, A. Scholey, Functional connectivity of the anterior and posterior hippocampus: Differential effects of glucose in younger and older adults. *Front. Aging Neurosci.* **12**, 8 (2020).

25. O. Tschritter, A. Fritsche, C. Thamer, M. Haap, F. Shirkavand, S. Rahe, H. Staiger, E. Maerker, H. Häring, M. Stumvoll, Plasma adiponectin concentrations predict insulin sensitivity of both glucose and lipid metabolism. *Diabetes* **52**, 239–243 (2003).

26. Y. Wu, B. Ehlert, A. A. Metwally, D. Perelman, H. Park, A. W. Brooks, F. Abbasi, B. Michael, A. Celli, C. Bejikian, E. Ayhan, Y. Lu, S. M. Lancaster, D. Hornburg, L. Ramirez, D. Bogumil, S. Pollock, F. Wong, D. Bradley, G. Gutjahr, E. S. Rangan, T. Wang, L. McGuire, P. Venkat Rangan, H. Ræder, Z. Shipony, D. Lipson, T. McLaughlin, M. P. Snyder,

Individual variations in glycemic responses to carbohydrates and underlying metabolic physiology. *Nat. Med.* **31**, 2232–2243 (2025).

27. R. Zangróniz, A. Martínez-Rodrigo, J. M. Pastor, M. T. López, A. Fernández-Caballero, Electrodermal activity sensor for classification of calm/distress condition. *Sensors* **17**, 2324 (2017).

28. L. Zeevi, M. Irani, C. Catana, L. Feldman Barrett, S. Atzil, Maternal dopamine encodes affective signals of human infants. *Soc. Cogn. Affect. Neurosci.* **17**, 503–509 (2022).

29. Y. Liu, S. Du, Psychological stress level detection based on electrodermal activity. *Behav. Brain Res.* **341**, 50–53 (2018).

30. W. Boucsein, *Electrodermal Activity* (Springer US, Boston, MA, 2012).

31. American Diabetes Association Professional Practice Committee, 2. Diagnosis and classification of diabetes: Standards of care in diabetes—2024. *Diabetes Care* **47**, S20–S42 (2024).

32. L. Beckes, J. A. Coan, Social baseline theory: The role of social proximity in emotion and economy of action. *Soc. Personal. Psychol. Compass* **5**, 976–988 (2011).

33. J. A. Coan, H. S. Schaefer, R. J. Davidson, Lending a hand: Social regulation of the neural response to threat. *Psychol. Sci.* **17**, 1032–1039 (2006).

34. T. D. Seeley, *The Wisdom of the Hive: The Social Physiology of Honey Bee Colonies* (Harvard Univ. Press, Cambridge, Mass, 1995).

35. R. Dunbar, Evolution of the social brain. *Science* **302**, 1160–1161 (2003).

36. R. I. M. Dunbar, The social brain hypothesis and its implications for social evolution. *Ann. Hum. Biol.* **36**, 562–572 (2009).

37. S. M. Burns, L. Tsoi, E. B. Falk, S. P. H. Speer, L. Mwilambwe-Tshilobo, D. I. Tamir, Interdependent minds: Quantifying the dynamics of successful social interactions. *Curr. Dir. Psychol. Sci.* , 09637214251323598 (2025).

38. T. H. Clutton-Brock, E. Huchard, Social competition and selection in males and females. *Philos. Trans. R. Soc. B. Biol. Sci.* **368**, 20130074 (2013).

39. P. Stockley, A. Campbell, Female competition and aggression: interdisciplinary perspectives. *Philos. Trans. R. Soc. B Biol. Sci.* **368**, 20130073 (2013).

40. J. B. Silk, S. C. Alberts, J. Altmann, Social bonds of female baboons enhance infant survival. *Science* **302**, 1231–1234 (2003).

41. J. B. Silk, J. C. Beehner, T. J. Bergman, C. Crockford, A. L. Engh, L. R. Moscovice, R. M. Wittig, R. M. Seyfarth, D. L. Cheney, The benefits of social capital: Close social bonds among female baboons enhance offspring survival. *Proc. R. Soc. B. Biol. Sci.* **276**, 3099–3104 (2009).

42. O. Schülke, J. Bhagavatula, L. Vigilant, J. Ostner, Social bonds enhance reproductive success in male macaques. *Curr. Biol.* **20**, 2207–2210 (2010).

43. D. I. Tamir, B. L. Hughes, Social rewards: From basic social building blocks to complex social behavior. *Perspect. Psychol. Sci.* **13**, 700–717 (2018).

44. T. Pradeu, Organisms or biological individuals? Combining physiological and evolutionary individuality. *Biol. Philos.* **31**, 797–817 (2016).

45. C. M. Child, The basis of physiological individuality in organisms. *Science* **43**, 511–523 (1916).

46. W. D. Hamilton, Altruism and related phenomena, mainly in social insects. *Annu. Rev. Ecol. Syst.* **3**, 193–232 (1972).

47. G. Roberts, Cooperation through interdependence. *Anim. Behav.* **70**, 901–908 (2005).

48. S. Atzil, W. Gao, I. Fradkin, L. F. Barrett, Growing a social brain. *Nat. Hum. Behav.* **2**, 624–636 (2018).

49. S. Cohen, T. A. Wills, Stress, social support, and the buffering hypothesis. *Psychol. Bull.* **98**, 310–357 (1985).

50. M. R. Gunnar, C. E. Hostinar, The social buffering of the hypothalamic–pituitary–adrenocortical axis in humans: Developmental and experiential determinants. *Soc. Neurosci.* **10**, 479–488 (2015).

51. B. N. Uchino, J. T. Cacioppo, J. K. Kiecolt-Glaser, The relationship between social support and physiological processes: A review with emphasis on underlying mechanisms and implications for health. *Psychol. Bull.* **119**, 488–531 (1996).

52. C. Shen, R. Zhang, J. Yu, B. J. Sahakian, W. Cheng, J. Feng, Plasma proteomic signatures of social isolation and loneliness associated with morbidity and mortality. *Nat. Hum. Behav.* **9**, 569–583 (2025).

53. N. Liang, S. J. Grayson, M. A. Kussman, J. N. Mildner, D. I. Tamir, In-person and virtual social interactions improve well-being during the COVID-19 pandemic. *Comput. Hum. Behav. Rep.* **15**, 100455 (2024).

54. H. Jarrell, J. B. Hoffman, J. R. Kaplan, S. Berga, B. Kinkead, M. E. Wilson, Polymorphisms in the serotonin reuptake transporter gene modify the consequences of social status on metabolic health in female rhesus monkeys. *Physiol. Behav.* **93**, 807–819 (2008).

55. J. E. Ho, M. G. Larson, R. S. Vasan, A. Ghorbani, S. Cheng, E. P. Rhee, J. C. Florez, C. B. Clish, R. E. Gerszten, T. J. Wang, Metabolite profiles during oral glucose challenge. *Diabetes* **62**, 2689–2698 (2013).

56. A. Bartolomucci, A. Cabassi, P. Govoni, G. Ceresini, C. Cero, D. Berra, H. Dadomo, P. Franceschini, G. Dell'Omo, S. Parmigiani, P. Palanza, Metabolic consequences and vulnerability to diet-induced obesity in male mice under chronic social stress. *PLOS ONE* **4**, e4331 (2009).

57. Y. C. Yang, T. Li, Y. Ji, Impact of social integration on metabolic functions: Evidence from a nationally representative longitudinal study of US older adults. *BMC Public Health* **13**, 1210 (2013).

58. N. Vaidya, A. F. Marquand, F. Nees, S. Siehl, G. Schumann, The impact of psychosocial adversity on brain and behaviour: An overview of existing knowledge and directions for future research. *Mol. Psychiatry* **29**, 3245–3267 (2024).

59. Y. C. Yang, C. Boen, K. Gerken, T. Li, K. Schorpp, K. M. Harris, Social relationships and physiological determinants of longevity across the human life span. *Proc. Natl. Acad. Sci. U.S.A.* **113**, 578–583 (2016).

60. J. T. Cacioppo, L. C. Hawkley, Social isolation and health, with an emphasis on underlying mechanisms. *Perspect. Biol. Med.* **46**, S39–S52 (2003).

61. C. H. Kroenke, L. D. Kubzansky, E. S. Schernhammer, M. D. Holmes, I. Kawachi, Social networks, social support, and survival after breast cancer diagnosis. *J. Clin. Oncol.* **24**, 1105–1111 (2006).

62. S. Cacioppo, J. T. Cacioppo, Decoding the invisible forces of social connections. *Front. Integr. Neurosci.* **6**, 51 (2012).

63. A. Vora, A. D. Nguyen, C. Spicer, W. Li, The impact of social isolation on health and behavior in *Drosophila melanogaster* and beyond. *Brain Sci. Adv.* **8**, 183–196 (2022).

64. N. J. Queen, W. Huang, S. Komatineni, A. G. Mansour, R. Xiao, L. A. Chrislip, L. Cao, Social isolation exacerbates diet-induced obesity and peripheral inflammation in young male mice under thermoneutrality. *iScience* **26**, 106259 (2023).

65. E. Kanitz, M. Tuchscherer, B. Puppe, A. Tuchscherer, B. Stabenow, Consequences of repeated early isolation in domestic piglets (*Sus scrofa*) on their behavioural, neuroendocrine, and immunological responses. *Brain Behav. Immun.* **18**, 35–45 (2004).

66. K. L. Bales, S. Hang, J. P. Paulus, E. Jahanfard, C. Manca, G. Jost, C. Boyer, R. Bern, D. Yerumyan, S. Rogers, S. L. Mederos, Individual differences in social homeostasis. *Front. Behav. Neurosci.* **17**, 1068609 (2023).

67. J. T. Cacioppo, L. C. Hawkley, G. J. Norman, G. G. Berntson, Social isolation. *Ann. N. Y. Acad. Sci.* **1231**, 17–22 (2011).

68. M. Jiang, L. Wang, H. Sheng, Mitochondria in depression: The dysfunction of mitochondrial energy metabolism and quality control systems. *CNS Neurosci. Ther.* **30**, e14576 (2024).

69. P. Fernandes, H. Nunes, T. A. Marinho, P. S. Barsanele, M. N. Moraes, M. de O. Poletini, Social isolation increases metabolic rate in the transition of light- to dark- phase and advances *Rev-erb*-α expression in brown adipose tissue to regulate daily rhythm of core body temperature in mice. *J. Photochem. Photobiol.* **16**, 100198 (2023).

70. H. Pourriyahi, N. Yazdanpanah, A. Saghazadeh, N. Rezaei, Loneliness: An immunometabolic syndrome. *Int. J. Environ. Res. Public Health* **18**, 12162 (2021).

71. R. E. Henriksen, T. Torsheim, F. Thuen, Loneliness, social integration and consumption of sugar-containing beverages: Testing the social baseline theory. *PLOS ONE* **9**, e104421 (2014).

72. M. N. Cramer, D. Gagnon, O. Laitano, C. G. Crandall, Human temperature regulation under heat stress in health, disease, and injury. *Physiol. Rev.* **102**, 1907–1989 (2022).

73. H. IJzerman, J. A. Coan, F. M. A. Wagemans, M. A. Missler, I. van Beest, S. Lindenberg, M. Tops, A theory of social thermoregulation in human primates. *Front. Psychol.* **6**, 464 (2015).

74. H. IJzerman, G. R. Semin, The thermometer of social relations: Mapping social proximity on temperature. *Psychol. Sci.* **20**, 1214–1220 (2009).

75. L. A. D. Campbell, P. J. Tkaczynski, J. Lehmann, M. Mouna, B. Majolo, Social thermoregulation as a potential mechanism linking sociality and fitness: Barbary macaques with more social partners form larger huddles. *Sci. Rep.* **8**, 6074 (2018).

76. H. IJzerman, L. Neyroud, R. Courset, M. Schrama, J. Post, T. M. Pronk, Socially thermoregulated thinking: How past experiences matter in thinking about our loved ones. *J. Exp. Soc. Psychol.* **79**, 349–355 (2018).

77. H. IJzerman, S. Lindenberg, İ. Dalğar, S. S. C. Weissgerber, R. C. Vergara, A. H. Cairo, M. V. Čolić, P. Dursun, N. Frankowska, R. Hadi, C. J. Hall, Y. Hong, C.-P. Hu, J. Joy-Gaba, D. Lazarević, L. B. Lazarević, M. Parzuchowski, K. G. Ratner, D. Rothman, S. Sim, C. Simão, M. Song, D. Stojilović, J. K. Blomster, R. Brito, M. Hennecke, F. Jaume-Guazzini, T. W. Schubert, A. Schütz, B. Seibt, J. H. Zickfeld, The human penguin project: Climate, social integration, and core body temperature. *Collabra Psychol.* **4**, 37 (2018).

78. L. Beckes, D. A. Sbarra, Social baseline theory: State of the science and new directions. *Curr. Opin. Psychol.* **43**, 36–41 (2022).

79. J. A. Coan, D. A. Sbarra, *Social Baseline Theory*: The social regulation of risk and effort. *Curr. Opin. Psychol.* **1**, 87–91 (2015).

80. C. R. Lee, A. Chen, K. M. Tye, The neural circuitry of social homeostasis: Consequences of acute versus chronic social isolation. *Cell* **184**, 1500–1516 (2021).

81. G. A. Matthews, K. M. Tye, Neural mechanisms of social homeostasis. *Ann. N. Y. Acad. Sci.* **1457**, 5–25 (2019).

82. P. Goldstein, I. Weissman-Fogel, G. Dumas, S. G. Shamay-Tsoory, Brain-to-brain coupling during handholding is associated with pain reduction. *Proc. Natl. Acad. Sci. U.S.A.* **115**, E2528–E2537 (2018).

83. M. Mikulincer, P. R. Shaver, Attachment orientations and emotion regulation. *Curr. Opin. Psychol.* **25**, 6–10 (2019).

84. M. R. Gunnar, B. Donzella, Social regulation of the cortisol levels in early human development. *Psychoneuroendocrinology* **27**, 199–220 (2002).

85. M. Gunnar, K. Quevedo, The neurobiology of stress and development. *Annu. Rev. Psychol.* **58**, 145–173 (2007).

86. J. Bowlby, *A Secure Base: Clinical Applications of Attachment Theory* (Psychology Press, 1988).

87. A. Stijovic, P. Forbes, E. Pronizius, A. Feneberg, G. Piperno, U. M. Nater, C. Lamm, G. Silani, Affective and social predictors of food consumption during the COVID-19 lockdown. *Biol. Psychiatry* **97**, 1002–1010 (2025).

88. G. Sproesser, H. T. Schupp, B. Renner, The bright side of stress-induced eating: Eating more when stressed but less when pleased. *Psychol. Sci.* **25**, 58–65 (2014).

89. T. Ein-Dor, J. A. Coan, A. Reizer, E. B. Gross, D. Dahan, M. A. Wegener, R. Carel, C. R. Cloninger, A. H. Zohar, Sugarcoated isolation: Evidence that social avoidance is linked to higher basal glucose levels and higher consumption of glucose. *Front. Psychol.* **6**, 492 (2015).

90. V. Chandra, I. Fetter-Pruneda, P. R. Oxley, A. L. Ritger, S. K. McKenzie, R. Libbrecht, D. J. C. Kronauer, Social regulation of insulin signaling and the evolution of eusociality in ants. *Science* **361**, 398–402 (2018).

91. P. K. Olszewski, A. Klockars, A. S. Levine, Oxytocin: A conditional anorexigen whose effects on appetite depend on the physiological, behavioural and social contexts. *J. Neuroendocrinol.* **28**, 10.1111/jne.12376 (2016).

92. P. K. Olszewski, K. Allen, A. S. Levine, Effect of oxytocin receptor blockade on appetite for sugar is modified by social context. *Appetite* **86**, 81–87 (2015).

93. Y. Park, B. P. Don, A. E. Mason, A. A. Prather, E. S. Epel, Daily social resources as a buffer against stress eating and its consequences. *Health Psychol.* **44**, 108–118 (2025).

94. M. B. Sokolowski, Social eating for stress. *Nature* **419**, 893–894 (2002).

95. M. de Bono, D. M. Tobin, M. W. Davis, L. Avery, C. I. Bargmann, Social feeding in *Caenorhabditis elegans* is induced by neurons that detect aversive stimuli. *Nature* **419**, 899–903 (2002).

96. M. Cohen, M. Abargil, M. Ahissar, S. Atzil, Social and nonsocial synchrony are interrelated and romantically attractive. *Commun. Psychol.* **2**, 57 (2024).

97. M. Djerassi, S. Ophir, S. Atzil, What is social about autism? The role of allostasis-driven learning. *Brain Sci.* **11**, 1269 (2021).

98. S. Atzil, M. Gendron, Bio-behavioral synchrony promotes the development of conceptualized emotions. *Curr. Opin. Psychol.* **17**, 162–169 (2017).

99. M. E. Carter, J. S. Borg, L. de Lecea, The brain hypocretins and their receptors: Mediators of allostatic arousal. *Curr. Opin. Pharmacol.* **9**, 39–45 (2009).

100. D. S. Quintana, A. J. Guastella, An allostatic theory of oxytocin. *Trends Cogn. Sci.* **24**, 515–528 (2020).

101. J. Schulkin, *Rethinking Homeostasis: Allostatic Regulation in Physiology and Pathophysiology* (MIT Press, 2002).

102. P. Sterling, Allostasis: A model of predictive regulation. *Physiol. Behav.* **106**, 5–15 (2012).

103. P. Sterling, Homeostasis vs allostasis: Implications for brain function and mental disorders. *JAMA Psychiatry* **71**, 1192–1193 (2014).

104. J. Schulkin, P. Sterling, Allostasis: A brain-centered, predictive mode of physiological regulation. *Trends Neurosci.* **42**, 740–752 (2019).

105. P. Sterling, S. Laughlin, Why an animal needs a brain. *Anim. Cogn.* **26**, 1751–1762 (2023).

106. I. R. Kleckner, J. Zhang, A. Touroutoglou, L. Chanes, C. Xia, W. K. Simmons, K. S. Quigley, B. C. Dickerson, L. F. Barrett, Evidence for a large-scale brain system supporting allostasis and interoception in humans. *Nat. Hum. Behav.* **1**, 0069 (2017).

107. J. E. Theriault, Y. Katsumi, H. M. Reimann, J. Zhang, P. Deming, B. C. Dickerson, K. S. Quigley, L. F. Barrett, It's not the thought that counts: Allostasis at the core of brain function. *Neuron* **113**, 4107–4133 (2025).

108. L. F. Barrett, W. K. Simmons, Interoceptive predictions in the brain. *Nat. Rev. Neurosci.* **16**, 419–429 (2015).

109. J. Zhang, D. Chen, P. Deming, T. Srirangarajan, J. E. Theriault, P. A. Kragel, L. Hartley, K. M. Lee, K. McVeigh, T. D. Wager, L. L. Wald, A. B. Satpute, K. S. Quigley, S. Whitfield-Gabrieli, L. F. Barrett, M. Bianciardi, Cortical and subcortical mapping of the human allostatic–interoceptive system using 7 Tesla fMRI. *Nat. Neurosci.* **28**, 2380–2391 (2025).

110. F. Faul, E. Erdfelder, A.-G. Lang, A. Buchner, G*Power 3: A flexible statistical power analysis program for the social, behavioral, and biomedical sciences. *Behav. Res. Methods* **39**, 175–191 (2007).

111. A. Lantagne, W. Furman, Romantic relationship development: The interplay between age and relationship length. *Dev. Psychol.* **53**, 1738–1749 (2017).

112. Accu-Chek UK & Ireland, Accu-Chek Instant Blood Glucose Meter; www.accu-chek.co.uk/products/meters/instant.

113. A. C. Meyer-Gerspach, L. Cajacob, D. Riva, R. Herzog, J. Drewe, C. Beglinger, B. K. Wölnerhanssen, Mechanisms regulating insulin response to intragastric glucose in lean and non-diabetic obese subjects: A randomized, double-blind, parallel-group trial. *PLOS ONE* **11**, e0150803 (2016).

114. K. F. Frøslie, J. Røislien, E. Qvigstad, K. Godang, J. Bollerslev, N. Voldner, T. Henriksen, M. B. Veierød, Shape information from glucose curves: Functional data analysis compared with traditional summary measures. *BMC Med. Res. Methodol.* **13**, 6 (2013).

115. O. Tschritter, A. Fritsche, F. Shirkavand, F. Machicao, H. Häring, M. Stumvoll, Assessing the shape of the glucose curve during an oral glucose tolerance test. *Diabetes Care* **26**, 1026–1033 (2003).

116. A. Tirosh, I. Shai, D. Tekes-Manova, E. Israeli, D. Pereg, T. Shochat, I. Kochba, A. Rudich, Normal fasting plasma glucose levels and type 2 diabetes in young men. *N. Engl. J. Med.* **353**, 1454–1462 (2005).

117. American Diabetes Association, Standards of medical care in diabetes—2011. *Diabetes Care* **34**, S11–S61 (2011).

118. M. Riddle, G. Umpierrez, A. DiGenio, R. Zhou, J. Rosenstock, Contributions of basal and postprandial hyperglycemia over a wide range of A1C levels before and after treatment intensification in type 2 diabetes. *Diabetes Care* **34**, 2508–2514 (2011).

119. EmbracePlus: The world's most advanced smartwatch for continuous health monitoring; https://www.empatica.com/embraceplus.

120. E. Hauser, UNIX time, UTC, and datetime: Jussivity, prolepsis, and incorrigibility in modern timekeeping. *Proc. Assoc. Inf. Sci. Technol.* **55**, 161–170 (2018).

121. BIOPAC Systems Inc., BIOPAC: Data Acquisition, Loggers, Amplifiers, Transducers, Electrodes; www.biopac.com/.

122. W. B. Mendes, Assessing autonomic nervous system activity. *Methods Soc. Neurosci.* **118**, 21 (2009).

123. K. S. Quigley, "Sympathetic nervous system" in *The Corsini Encyclopedia of Psychology*, I. B. Weiner, W. E. Craighead, Eds. (Wiley, ed. 1, 2010), pp. 1–2.

124. D. C. Fowles, M. J. Christie, R. Edelberg, W. W. Grings, D. T. Lykken, P. H. Venables, Publication recommendations for electrodermal measurements. *Psychophysiology* **18**, 232–239 (1981).

125. D. T. Lykken, P. H. Venables, Direct measurement of skin conductance: A proposal for standardization. *Psychophysiology* **8**, 656–672 (1971).

126. B. G. Wallin, Sympathetic nerve activity underlying electrodermal and cardiovascular reactions in man. *Psychophysiology* **18**, 470–476 (1981).

127. C. Setz, B. Arnrich, J. Schumm, R. La Marca, G. Tröster, U. Ehlert, Discriminating stress from cognitive load using a wearable EDA device. *IEEE Trans. Inf. Technol. Biomed.* **14**, 410–417 (2010).

128. A. Klimek, I. Mannheim, G. Schouten, E. J. M. Wouters, M. W. H. Peeters, Wearables measuring electrodermal activity to assess perceived stress in care: A scoping review. *Acta Neuropsychiatr.* **37**, e19 (2023).

129. BIOPAC, AcqKnowledge Data Acquisition and Analysis Software - Win: ACK100W, ACK100M: Research; www.biopac.com/product/acqknowledge-software/.

130. H. F. Posada-Quintero, K. H. Chon, Innovations in electrodermal activity data collection and signal processing: A systematic review. *Sensors* **20**, 479 (2020).

131. S. Föll, M. Maritsch, F. Spinola, V. Mishra, F. Barata, T. Kowatsch, E. Fleisch, F. Wortmann, FLIRT: A feature generation toolkit for wearable data. *Comput. Methods Programs Biomed.* **212**, 106461 (2021).

132. K. Sharma, C. Castellini, E. L. van den Broek, A. Albu-Schaeffer, F. Schwenker, A dataset of continuous affect annotations and physiological signals for emotion analysis. *Sci. Data* **6**, 196 (2019).

133. K. S. Erer, Adaptive usage of the Butterworth digital filter. *J. Biomech.* **40**, 2934–2943 (2007).

134. M. Trapanotto, F. Benini, M. Farina, D. Gobber, V. Magnavita, F. Zacchello, Behavioural and physiological reactivity to noise in the newborn. *J. Paediatr. Child Health* **40**, 275–281 (2004).

135. UM-16Rev.2.0 20201020-E4_user_manual.pdf, Powered by Box; https://www.empatica.com/blog/decoding-wearable-sensor-signals-what-to-expect-from-your-e4-data/.

136. C. T. Ekstrøm, MESS: Miscellaneous Esoteric Statistical Scripts (2012); https://doi.org/10.32614/CRAN.package.MESS.

137. G. B. Spanier, Measuring dyadic adjustment: New scales for assessing the quality of marriage and similar dyads. *J. Marriage Fam.* **38**, 15–28 (1976).

138. R. E. Kass, A. E. Raftery, Bayes factors. *J. Am. Stat. Assoc.* **90**, 773–795 (1995).

139. American Diabetes Association Professional Practice Committee, 2. Classification and diagnosis of diabetes: Standards of medical care in diabetes—2022. *Diabetes Care* **45**, S17–S38 (2022).
